# Value of AI in Critical Care Using Real-World Evidence on Intensive Care Unit Mortality Prediction: Cost-Utility Analysis

**DOI:** 10.2196/93466

**Published:** 2026-08-18

**Authors:** Gyeong Min Lee, Joo-Yun Won, Eun Young Cho, Ji-Hyun Kim, Kyung Soo Chung, Kwang Joon Kim, Seok Jin Yun, Hyun Jun Lee, Jae-Hyun Kim

**Affiliations:** 1 Research Institute for Healthcare Policy, Dankook University, Dandae-ro 119, Cheonan, Chungcheongnam-do, 31116, Republic of Korea, 82 10-8438-8353; 2 AITRICS Corp, Seoul, Republic of Korea; 3 Division of Pulmonology, Department of Internal Medicine, Institute for Innovation in Digital Healthcare, Severance Hospital, Yonsei University College of Medicine, Seoul, Republic of Korea; 4 Department of Health Administration, College of Public Health Sciences, Dankook University, Cheonan, Chungcheongnam-do, Republic of Korea

**Keywords:** artificial intelligence, intensive care unit, mortality prediction, cost-effectiveness, Markov model, health economics, decision support systems, real-world data

## Abstract

**Background:**

AI-based models for predicting mortality have shown potential for intensive care unit (ICU) patients, but evidence regarding their cost-effectiveness remains limited.

**Objective:**

This study aimed to evaluate the population-level cost-utility of an AI-based mortality prediction strategy activated during ICU admission in Korea.

**Methods:**

A lifetime Markov model followed a hypothetical cohort of Korean adults from age 19 years in the general-population state to capture ICU admissions, including recurrent ICU admissions, occurring over the lifetime horizon. AI-based mortality risk monitoring and its implementation cost were applied only when an individual entered the ICU state. Model inputs were derived from national claims data, published utility estimates, and cost sources. Outcomes were expressed as incremental cost-effectiveness ratios (ICERs) in Korean won (KRW) per quality-adjusted life year (QALY). Deterministic and probabilistic sensitivity analyses were conducted.

**Results:**

The AI-assisted strategy yielded an ICER of 8.37 million KRW (approximately US $6200) per QALY, below the societal willingness-to-pay (WTP) threshold of 40 million KRW (approximately US $29,600) per QALY in Korea. The incremental costs and QALYs were lifetime expected values per member of the general-population starting cohort rather than outcomes per directly monitored ICU admission. Probabilistic sensitivity analysis showed an 83.94% probability of cost-effectiveness at the threshold. One-way sensitivity analysis identified the true-positive rate, AI implementation cost, and postdischarge well-state utility value as the most influential parameters. Additional claims-based analyses indicated that ICU transition pathways and patient age were strongly associated with survival outcomes.

**Conclusions:**

Under modeled assumptions, AI-assisted ICU mortality prediction showed potential cost-effectiveness compared with usual care in the Korean critical care setting. These findings should be interpreted as decision-analytic estimates rather than direct evidence that the AI system reduces ICU mortality in real patients. Prospective real-world evaluations are needed to confirm clinical effectiveness and implementation value.

## Introduction

In recent years, technology-driven approaches have been actively explored to improve outcomes for patients in intensive care units (ICUs) and to promote efficient allocation of health care resources. ICUs provide intensive treatment for patients with severe illnesses, yet mortality rates remain high, and the associated treatment costs are substantially greater than those of general wards (GWs) [1,2]. Consequently, approaches that predict clinical deterioration early and support timely management have garnered increasing attention in clinical practice [3].

Within this context, AI prediction models have emerged as a promising tool for identifying high-risk patients through the integration of vital signs, medical history, and treatment data. Several AI-based early warning systems for clinical deterioration have been implemented in hospital settings, including ICU monitoring and GW surveillance systems [4,5]. In this study, the AI model refers to a machine learning (ML)–based clinical decision support system (CDSS) that estimates in-ICU mortality risk using routinely collected patient data and provides risk stratification information to clinicians.

Internationally, numerous AI prediction models have been developed to predict ICU mortality, showing high accuracy in identifying high-risk patients at an early stage [5]. However, most existing studies have focused primarily on algorithmic performance rather than on downstream clinical or economic outcomes.

In contrast, this study specifically evaluates the cost-effectiveness of AI-assisted mortality prediction within ICU settings, where patients are already critically ill and resource use is intensive. By analyzing long-term costs and quality-adjusted life years (QALYs), this study investigates whether the application of AI-based mortality prediction has the potential to improve both clinical outcomes and economic efficiency in critical care settings.

AI prediction models entail development, installation, and operational costs but can potentially yield economic value and cost-effectiveness by supporting earlier clinical decision-making and optimizing resource use [6,7]. Evaluating their economic value provides practical insights for health care policymakers and institutions seeking to balance innovation with sustainability.

In Korea, overall health care expenditure under the National Health Insurance system has been rapidly increasing, with ICU-related medical costs accounting for a considerable share [8,9]. In resource-constrained environments, the selection of cost-effective technologies and prioritization of health care strategies are essential for sustaining the health care system. Health economic evaluation has become a key policy instrument to support these choices, with cost-utility analysis using QALYs serving as a standardized method for comparing the relative efficiency of medical technologies and programs [3].

Accordingly, this study aimed to evaluate the population-level cost-effectiveness of an AI-assisted mortality prediction strategy activated during ICU admission using simulation-based modeling and empirical evidence derived from national claims data. Rather than analyzing real-time implementation in clinical settings, the study assessed whether such AI models could operate cost-effectively under real-world health care conditions in Korea.

A Markov model was used to quantitatively assess the economic feasibility of the AI-based approach, and sensitivity analyses were conducted to validate cost-effectiveness under a unified cost scenario. This design was intended to ensure the findings offer practical insights into health care policy decision-making.

This study extends previous research in several ways. First, it moves beyond simple evaluations of predictive accuracy to comprehensively analyze the costs and outcomes of AI prediction models in practical health care contexts. Second, it translates the effects of AI into lifetime costs and health utilities, presenting concrete evidence that can inform health care policy. Third, it enhances the robustness and policy relevance of the results through both deterministic and probabilistic sensitivity analyses, demonstrating the potential for AI-based prediction models to contribute to improved care quality and resource efficiency, based on modeled estimates.

## Methods

### Study Design

This study evaluated the cost-effectiveness of an AI prediction model in the ICU using a decision-analytic framework. A state-transition Markov model was constructed as the primary analytic approach to compare long-term costs and health outcomes, expressed as QALYs, between AI-assisted care and usual care.

To inform model inputs with real-world evidence, supplementary empirical analyses were conducted using the National Health Insurance Service–National Sample Cohort (NHIS-NSC) database. These analyses were used to derive transition probabilities, health care use patterns, and cost parameters, and to provide contextual descriptive information on ICU patient trajectories.

By integrating simulation-based modeling with national claims data, the study aimed to assess the potential clinical and economic value of AI prediction models in the Korean health care system. Sources and derivation methods for major model parameters, including diagnostic performance inputs, are summarized in Multimedia Appendix 1. This study was conducted and reported in accordance with the CHEERS-AI (Consolidated Health Economic Evaluation Reporting Standards for Interventions That Use Artificial Intelligence). A completed CHEERS-AI checklist is provided in Checklist 1.

### Model Structure

**Figure 1.**
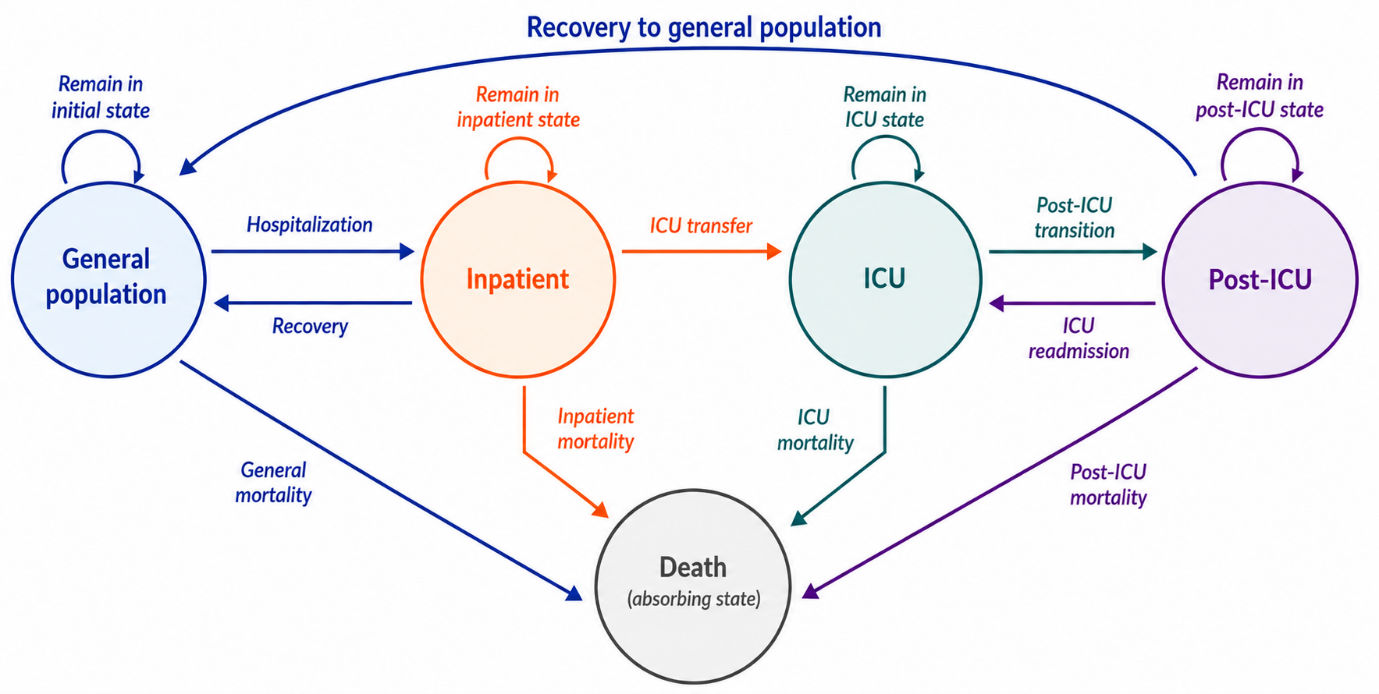
Markov model structure for intensive care unit (ICU) mortality prediction and post-ICU transition.

A state-transition Markov model was developed to represent major clinical pathways related to hospitalization, ICU care, post-ICU inpatient recovery, and death. The model included 5 mutually exclusive health states: general population, inpatient, ICU, post-ICU inpatient recovery, and death. Death was modeled as an absorbing state. Individuals could remain in their current nondeath state, transition from the general population to inpatient care, transfer from inpatient care to ICU, transition from ICU to post-ICU inpatient recovery, experience ICU readmission from the post-ICU state, recover to the general population, or die according to state-specific mortality probabilities. Direct recovery from ICU back to the initial inpatient state was not modeled; patients surviving ICU care and requiring continued inpatient management entered the post-ICU inpatient recovery state. The model used a 1-month cycle length and a lifetime horizon (Figure 1).

Diagnostic performance parameters were informed by implementation summary data from the deployed AI early warning system under the selected alert threshold. These inputs were intended to represent early identification of high-risk clinical deterioration rather than disease-specific diagnosis alone. Additional details are provided in Multimedia Appendix 2.

The modeled population was a hypothetical cohort of Korean adults entering the model at age 19 years in the general-population state. Individuals were followed over a lifetime horizon and could subsequently experience hospitalization, ICU admission, post-ICU recovery, and recurrent ICU admission. The general-population starting state was adopted to allow the ICU-activated AI strategy to operate across the entire lifetime horizon and to capture both initial and repeated ICU admissions, rather than restricting the analysis to a single index ICU stay. The AI-assisted and usual-care strategies differed only upon entry into the ICU state, when AI-based mortality risk monitoring was activated in the AI-assisted strategy. Accordingly, the model estimated the cumulative population-level lifetime expected costs and QALYs associated with all eligible ICU-monitoring episodes rather than outcomes conditional on a single index ICU admission.

### Clinical Workflow and Safety Assumptions for AI Alerts

The AI prediction model was conceptualized as a clinician-facing CDSS rather than an autonomous diagnostic or treatment system [10]. In the modeled workflow, AI-generated high-risk alerts were assumed to notify clinicians of increased predicted risk of deterioration or mortality and to prompt clinician review, bedside reassessment, closer monitoring, and potential escalation of care when clinically appropriate [11,12]. The model did not assume that AI alerts directly caused treatment changes, ICU transfer, invasive procedures, or mortality reduction without clinician interpretation and action [10]. For true-positive alerts, clinicians were assumed to recognize high-risk patients earlier and consider appropriate actions according to local protocols [13]. For false-positive alerts, clinical reassessment was assumed without automatic intervention, acknowledging the potential for increased workload and alert fatigue [12]. Specificity was reported to characterize the diagnostic performance of the AI system; however, false-positive alerts were not modeled as a separate economic branch. No separate per-alert costs were assigned for additional chart review, bedside reassessment, diagnostic testing, or treatment. The aggregate AI implementation cost reflected routine system operation and workflow integration but did not explicitly quantify the marginal clinical workload associated with each false-positive alert. For false-negative cases, usual care and routine monitoring were assumed to continue because the AI system was not intended to replace standard clinical surveillance [14]. Clinicians retained final responsibility for interpretation and management decisions, and implementation was assumed to require local alert thresholds, clinician training, documentation of alert response, periodic review of false-positive alerts and missed high-risk cases, and monitoring for automation bias [15]. These assumptions were used to clarify the modeled human-AI interaction pathway and were not interpreted as directly observed implementation outcomes.

### AI-Specific Reporting and Implementation Assumptions

The AI model whose performance parameters were applied in this economic evaluation was VitalCare-Mortality Score (VC-MORS; AITRICS Corp), a deep learning–based early warning system for ICU mortality risk prediction. VC-MORS is approved by the Korean Ministry of Food and Drug Safety and is intended for ICU use. The performance parameters were derived from a real-world implementation study [16] in which the pretrained model was deployed without retraining or recalibration during the study period; therefore, the reported performance reflects a single fixed version of the algorithm evaluated in that source study. Although the commercial product may be updated through subsequent version releases and its risk scores are refreshed whenever new electronic medical record data are recorded, the parameter estimates used in this analysis corresponded to the fixed version evaluated in the source study.

VC-MORS primarily uses routinely measured vital signs and age as core inputs, with additional variables incorporated when available in the electronic medical record. The system generates a risk score ranging from 0 to 100, indicating the risk of mortality within the next 6 hours. Risk scores and alerts are presented to attending health care providers, including ICU nurses and physicians, through the VitalCare monitoring interface, which displays each patient’s current risk score, score trends over time, alarm history, and relevant single-parameter values. The reported performance of VC-MORS used in this economic evaluation was based on ICU patients only, not on the broader hospitalized population or GW patients. The model architecture has been described previously [17], but further technical implementation details beyond the published model architecture were not fully disclosed because they are proprietary information of AITRICS Corp.

### Clinical Effectiveness Inputs

Age-stratified transition probabilities for the Markov model were derived from the National Health Insurance Service (NHIS) claims database (2008‐2019). Because direct evidence quantifying mortality reduction attributable to ICU AI early warning systems remains limited, the effect of AI was modeled as an indirect system-level benefit pathway. Specifically, AI-based risk prediction was assumed to support earlier clinical recognition, escalation of monitoring, and timely intervention. Based on prior literature evaluating intensivist-supported ICU care and tele-ICU surveillance models, a mortality hazard ratio (HR) of 0.85 (95% CI 0.83‐0.89) was applied as a pragmatic proxy estimate [18-20].

The diagnostic-performance and mortality-reduction parameters were linked sequentially in the economic model. At each model cycle, ICU patients were probabilistically classified as true positive, with a probability equal to the sensitivity of 0.551, or false negative, with a probability equal to 1 − sensitivity, or 0.449. These probabilities were derived from the reference implementation study of the deployed AI early warning system summarized in Multimedia Appendix 2.

For true-positive cases, the high-risk alert was assumed to prompt earlier clinician recognition, bedside reassessment, closer monitoring, and clinically appropriate action. The mortality HR of 0.85 was applied exclusively to the ICU-state mortality transition probability of this subgroup, representing the assumed downstream effect conditional on correct identification and clinician-directed response. False-negative patients retained the unadjusted usual-care ICU mortality probability because no alert was generated.

Formally, the ICU mortality probability under the AI-assisted strategy was calculated as


$$\begin{array}{l} p(death|AI)=sensitivity\times [p(death|usual\;care)\times HR]+\\ \left(1-sensitivity)\times p(death|usual\;care\right) \end{array}$$

This formulation did not interpret the HR as a direct treatment effect of the algorithm itself. Rather, it represented a conditional system-level effect that could occur when a correct AI alert was translated into timely clinical recognition and action.

The discharge rate was estimated using Health Insurance Review and Assessment Service (HIRA) health care use statistics (2018‐2023). Age-specific admission rates and background mortality were obtained from national population and mortality statistics (Table 1).

**Table 1.** Input parameters for the cost-utility analysis of AI-assisted ICU^a^ mortality prediction.

Parameter	Base-case value, mean (SD)	Range	Distribution	Data source
Diagnostic accuracy				
True positive	0.551 (0.110)	0.335‐0.767	PERT^b^	Kim et al (2025) [16] (Multimedia Appendix 2)
False negative	0.449 (0.090)	0.273‐0.625	PERT	Kim et al (2025) [16] (Multimedia Appendix 2)
Mortality reduction (HR^c^)	0.850 (0.170)	0.830‐0.890	Log-normal	Oh and Song (2023) [20]
AI costs per monitored admission (KRW^d^)	1,200,000 (240,000)^e^	729,600‐1,670,400^f^	Gamma	Developer-provided assumption-based estimate, informed by Korean reimbursement rates for digital health care–based inpatient monitoring
QALY^g^ per 1-month cycle				
ICU	0.056 (0.011)	0.034‐0.0773	PERT	Cuthbertson et al (2010) [21]
GW^h^	0.066 (0.014)	0.042‐0.0833	PERT	Cuthbertson et al (2010) [21]
Postdischarge well	0.078 (0.017)	0.0507‐0.083	PERT	Model assumption: full monthly cycle utility for postdischarge survival; background mortality applied separately
Discharge probability	0.771 (0.154)	0.469‐1.000	PERT	Health Insurance Review and Assessment Service, Healthcare Service Utilization, 2023, and National Health Insurance Service and Health Insurance Review and Assessment Service, National Health Insurance Statistical Yearbook, 2023 [22,23], calculated as the mean discharge-to-admission ratio for 2018-2023.
Admission rate	Age-specific rate	—^i^	Beta	2023 National Health Insurance Statistical Yearbook and Ministry of the Interior and Safety resident registration population statistics [23,24], calculated as age-specific inpatient treatment persons divided by the corresponding population and converted to monthly probabilities
Background mortality	Age-specific rate	—	Beta	2023 Complete Life Table, Statistics Korea [25], converted from annual to monthly mortality probabilities

a ICU: intensive care unit.

b PERT: Program Evaluation and Review Technique.

c HR: hazard ratio.

d KRW: Korean won.

e Approximately US $890 (≈ US $178).

f Approximately US $540‐$1237.

g QALY: quality-adjusted life year.

h GW: general ward.

i Not applicable because age-specific values were applied rather than a single overall parameter value.

### Cost Input

Direct medical costs were estimated from the Korean NHIS claims database and reflect reimbursed expenditures within the Korean health care system. Age-stratified costs for ICU admission and GW hospitalization were incorporated into the model.

The AI implementation cost was defined as an aggregate allocated cost per monitored ICU admission, applied once per admission for the period of ICU monitoring, rather than as a separately itemized charge for each cost component. The base-case value was set at Korean won (KRW) 1,200,000 (approximately US $890) per monitored admission. This value was a developer-provided, assumption-based estimate because no reimbursement fee currently exists for AI-based patient monitoring in Korea. Therefore, this estimate was set with reference to the existing Korean reimbursement rates for a digital health care–based continuous monitoring solution used in GWs, including remote cardiac monitoring (EX871, KRW 44,287 [approximately US $33] per patient-day), transcutaneous oxygen saturation monitoring (E7230, KRW 9971 [approximately US $7] per patient-day), and bedside electrocardiographic monitoring (E6544, KRW 18,803 [approximately US $14] per patient-day), totaling approximately KRW 73,061 (approximately US $54) per patient-day. These rates were used as the closest valuation reference available, rather than as fees directly applicable to AI-based monitoring. The final base-case value was treated as an aggregate per-admission implementation estimate rather than a directly reimbursed per-day fee, and it was not intended to imply a fixed ICU length of stay.

The AI implementation cost was applied once for each monitored ICU admission in the AI-assisted strategy, including a recurrent ICU admission after transition from the post-ICU state. The general-population starting structure was adopted so that AI monitoring and its associated cost could be activated whenever an eligible ICU admission occurred over the lifetime horizon rather than being restricted to a single index ICU stay. Therefore, the expected AI cost per starting cohort member was determined by the probability, timing, and recurrence of ICU admissions and was discounted over the lifetime horizon. Thus, following discharge from an ICU monitoring episode, whether to post-ICU inpatient recovery or subsequently to the general population, any later eligible ICU admission was treated as a new VC-MORS monitoring episode, and the full KRW 1,200,000 (approximately US $890) implementation cost was applied again.

The aggregate AI cost was intended to reflect the per-admission share of system-level costs required to implement and operate the AI early warning system in ICU settings, including software licensing, server and infrastructure deployment, maintenance, technical support, staff training, workflow integration, and operational management. The component categories and allocation assumptions are summarized in Multimedia Appendix 3. Separately itemized commercial estimates for these components were not available; therefore, arbitrary component-level prices were not imputed. The base-case value was assigned an SD of KRW 240,000 (approximately US $178) and a range of KRW 729,600‐1,670,400 (approximately US $540‐$1237). This parameter was modeled using a Gamma distribution and varied in deterministic sensitivity analysis, probabilistic sensitivity analysis, and threshold analysis.

Age-specific hospitalization costs were separately applied. ICU admission costs ranged from KRW 8,509,577 to KRW 11,312,502 (approximately US $6300‐$8380), and GW costs ranged from KRW 1,154,666 to KRW 1,671,863 (approximately US $855‐$1240), depending on age group (Table 2). All costs were expressed in 2023 KRW. For international comparability, selected values are additionally presented in US $ using the 2023 average exchange rate of KRW 1350=US $1.

**Table 2.** Age-specific transition probabilities and medical costs^a^.

Age group (years)	Transition probability, mean (SD)				Medical cost, mean (SD)	Transition probability, mean (SD)				Medical cost, mean (SD)
	ICU^b^→GW^c^		ICU→death		General ward	GW→ICU		GW →death		ICU
19‐29	0.4628 (0.0926)		0.1022 (0.0204)		1,456,071 (291,214)	0.0001 (0.0000)		0.0047 (0.0009)		9,389,534 (1,877,907)
30‐39	0.4518 (0.0904)		0.1661 (0.0332)		1,522,857 (304,571)	0.0002 (0.0000)		0.0086 (0.0017)		10,963,960 (2,192,792)
40‐49	0.4413 (0.0883)		0.2346 (0.0469)		1,577,969 (315,594)	0.0003 (0.0001)		0.0179 (0.0036)		8,804,303 (1,760,861)
50‐59	0.4348 (0.0870)		0.2637 (0.0527)		1,653,276 (330,655)	0.0005 (0.0001)		0.0333 (0.0067)		9,901,725 (1,980,345)
60‐69	0.4238 (0.0848)		0.3462 (0.0692)		1,671,863 (334,373)	0.0006 (0.0001)		0.0774 (0.0155)		11,312,502 (2,262,500)
70‐79	0.3815 (0.0763)		0.5350 (0.1070)		1,472,711 (294,542)	0.0009 (0.0002)		0.2047 (0.0409)		9,990,410 (1,998,082)
≥80	0.3168 (0.0634)		0.7071 (0.1414)		1,154,666 (230,933)	0.0017 (0.0003)		0.4049 (0.0810)		8,509,577 (1,701,915)

a This table presents age-stratified transition probabilities between intensive care unit, general ward, and death health states, as well as age-specific hospitalization costs used in the Markov model. Transition probabilities and costs were estimated from customized National Health Insurance Service claims data for Korean adult inpatients. Costs are expressed in 2023 Korean won. Transition probabilities were modeled using Beta distributions, and medical costs were modeled using Gamma distributions.

b ICU: intensive care unit.

c GW: general ward.

### Utility Values

Health outcomes were measured as QALYs, with utility weights assigned to each Markov health state. Utility values for ICU admission, GW hospitalization, and postdischarge survival were derived from previously published studies of critically ill adults and ICU survivors reporting health-related quality-of-life outcomes, primarily Cuthbertson et al [21]. These source populations were considered clinically comparable to the target population because they involved patients who required intensive care and experienced post-ICU recovery. The monthly utility weight used in the deterministic base-case analysis was 0.056 for the ICU state, 0.066 for the GW state, 0.078 for the postdischarge well state, and 0.000 for death.

The postdischarge well state represents survival after hospital discharge rather than full restoration to age-matched population health. For the PERT-distributed utility parameters, the nominal values of 0.0683 for the GW state and 0.0833 for the postdischarge well state were used as modal inputs, whereas the corresponding distributional means of 0.0664 and 0.0779, respectively, were used as the deterministic base-case values. Where annual utility estimates were reported in the source literature, they were converted to cycle-specific monthly values to align with the 1-month Markov cycle. Reduced utility after critical illness was assumed to reflect persistent physical disability, cognitive impairment, and psychological sequelae commonly observed among ICU survivors [21].

Costs and QALYs were discounted at an annual rate of 4.5% in accordance with Korean health economic evaluation practice. Because the Markov model used monthly cycles, the annual discount rate was converted to a cycle-specific monthly discount factor and applied consistently to both costs and QALYs in the base-case analysis. Scenario analyses were conducted using alternative assumptions to assess the robustness of the results.

### Sensitivity Analysis

To evaluate parameter uncertainty, both deterministic sensitivity analysis (DSA) and probabilistic sensitivity analysis (PSA) were conducted. In the DSA, 1-way sensitivity analyses were performed across prespecified parameter ranges, and results were summarized using a tornado diagram to identify the variables with the greatest influence on the incremental cost-effectiveness ratio (ICER).

In the PSA, a Monte Carlo simulation with 100,000 iterations was conducted using probability distributions assigned to all key model parameters based on reported SDs or plausible ranges. Results were summarized using an incremental cost-effectiveness scatterplot and a cost-effectiveness acceptability curve (CEAC). To assess structural and clinical uncertainty, additional scenario analyses were performed using more conservative mortality benefit assumptions (HRs of 0.90 and 0.95) and alternative analytic horizons (5-year and 10-year). These results are reported in Multimedia Appendices 4 and 5.

Additional post-ICU burden scenario analyses were conducted as supplementary stress tests to address uncertainty related to simplified post-ICU trajectories. The base-case model included ICU readmission through the transition from the post-ICU state back to the ICU state. However, other recurrent post-ICU pathways, including non-ICU rehospitalization, post-ICU complications, rehabilitation, long-term care, and excess postdischarge mortality, were not modeled as separate health states because these pathways could not be identified with sufficient clinical granularity from the available claims-based model inputs. Therefore, these additional post-ICU pathways were evaluated through conservative scenario assumptions rather than incorporated as separate base-case health states.

Specifically, post-ICU quality of life was reduced to 95%, 90%, 85%, and 80% of the base-case value. In parallel, recurrent hospitalization or ICU-transition risk and post-ICU mortality risk were increased by 5%, 10%, 15%, and 20%. These scenarios were intended to reflect progressively worse recurrent post-ICU burden, including lower quality of life, increased rehospitalization or ICU readmission risk, and higher post-ICU mortality. These analyses were considered supplementary and were not interpreted as base-case results. The results are reported in Multimedia Appendix 6.

### Key Outcome Measures

The primary outcome measure of this study was the ICER, defined as the difference in costs between the AI-assisted care scenario and the standard care scenario divided by the difference in QALYs:


$$ICER=\frac{{Cost}_{AI}-{Cost}_{Control}}{{QALY}_{AI}-{QALY}_{Control}}$$

In addition, the willingness-to-pay (WTP) threshold was set at 40 million KRW per QALY, in line with national recommendations, and the net monetary benefit (NMB) was calculated accordingly. All analyses were performed in accordance with national and international guidelines for health economic evaluation [26]. Costs and QALYs were discounted at an annual rate of 4.5%, consistent with Korean health economic evaluation practice. Because the Markov model used monthly cycles, the annual discount rate was converted to a monthly cycle-specific discount factor and applied to both costs and QALYs.

### Use of National Claims Data

The NHIS-NSC was used to derive key model inputs, including transition probabilities, health care use patterns, and age-stratified cost estimates based on observed real-world data (REQ202400488-002). These empirically derived parameters were incorporated into the Markov model to better reflect clinical pathways and resource use within the Korean health care system. Additional descriptive analyses of patient trajectories and outcomes are provided in Multimedia Appendices 7–10.

A total of 214,005,418 inpatient episodes from adults aged ≥19 years (2008‐2019) were screened. After duplicate removal, application of a 1-year washout period, and inpatient episode construction, 101,366,575 inpatient episodes remained, including 70,998 ICU admissions, as shown in Multimedia Appendix 11.

### Ethical Considerations

This study was approved by the Institutional Review Board of Dankook University Hospital (IRB number DKUH202308010002). The requirement for informed consent was waived due to the use of anonymized secondary claims data. The Markov model was based on secondary data analysis and did not involve additional ethical considerations beyond the approved use of anonymized claims data.

### Data Access and Analytic Independence

Access to the NHIS claims data was restricted to authorized academic investigators under the approved data-use process. AITRICS Corp and AITRICS-affiliated authors did not have access to the NHIS claims data. The health economic model code and analytic files were developed and maintained by the academic research team. The sponsor did not control the analytic code, model assumptions, statistical analyses, or interpretation of the findings. No proprietary algorithm, confidential company-owned dataset, or patient-level commercial implementation data was used directly in the health economic model.

## Results

### Base-Case Analysis

The primary cost-utility results are presented in Table 3. The AI implementation cost was KRW 1,200,000 (approximately US $890) per monitored ICU admission and was applied once for each eligible ICU admission in the AI-assisted strategy. Because the analysis began with a general-population cohort at age 19 years, the model captured ICU admissions, including recurrent ICU admissions, occurring over the lifetime horizon. The resulting AI costs were therefore weighted by the probability, timing, and recurrence of ICU admission and were discounted over the lifetime horizon. The incremental cost of KRW 619 (approximately US $0.46) represents the lifetime expected incremental cost per member of the starting cohort rather than the cost difference for one directly monitored ICU admission. Accordingly, VC-MORS costs accumulated across repeated ICU admission-discharge-readmission episodes, with the full per-admission cost applied whenever monitoring was reinitiated at a new eligible ICU admission.

**Table 3.** Base-case cost-utility results for AI-assisted ICU^a^ mortality prediction versus usual care^b^.

Strategy starting age 19 years	Cost (KRW^c^)	Incremental cost (KRW)	Effectiveness (QALYs^d^)	Incremental effectiveness (QALYs)	ICER^e^ (KRW/QALY)	NMB^f^ (KRW)
AI implementation cost per monitored admission (1,200,000 KRW)						
Treatment as usual	2,746,228	—^g^	20.669723	—	—	824,042,703
AI prediction model	2,746,847	619	20.669797	0.000074	8,370,695	824,045,041

a ICU: intensive care unit.

b The base-case analysis used a Markov model from the Korean payer perspective and assumed a starting age of 19 years and an AI implementation cost of Korean won (KRW) 1,200,000 per monitored admission. Costs are presented in 2023 KRW. The incremental cost-effectiveness ratio was calculated as incremental cost divided by incremental quality-adjusted life years. Net monetary benefit was calculated using a willingness-to-pay threshold of KRW 40 million per quality-adjusted life year. Costs and quality-adjusted life years are reported as lifetime expected values per member of the general-population starting cohort. The AI implementation cost of KRW 1,200,000 was applied once for each monitored intensive care unit admission occurring in the AI-assisted strategy, including a recurrent intensive care unit admission. Because the model followed a general-population cohort over a lifetime horizon, the reported incremental cost reflects the probability, timing, and recurrence of intensive care unit admissions and should not be interpreted as the cost difference for a single monitored intensive care unit admission.

c KRW: Korean won.

d QALY: quality-adjusted life year.

e ICER: incremental cost-effectiveness ratio.

f NMB: net monetary benefit.

g Not applicable because incremental cost, incremental effectiveness, and the ICER are not applicable to the reference strategy.

Total expected costs were KRW 2,746,228 (approximately US $2034) for usual care and KRW 2,746,847 (approximately US $2035) for the AI-assisted strategy, corresponding to an incremental cost of KRW 619 (approximately US $0.46). Total effectiveness was 20.669723 and 20.669797 QALYs, respectively, yielding an incremental gain of 0.000074 QALYs and an ICER of KRW 8,370,695 (approximately US $6200) per QALY. The NMB analysis also showed an incremental gain of KRW 2338 (approximately US $1.73) versus usual care. Although the incremental health benefit was modest, the AI-assisted strategy was cost-effective under modeled base-case assumptions.

Workflow and safety assumptions for AI alerts were summarized in Multimedia Appendix 12. These assumptions were used to clarify the modeled human-AI interaction pathway and were not interpreted as directly observed workflow outcomes.

### Sensitivity Analysis: Probabilistic Sensitivity Analysis

**Figure 2.**
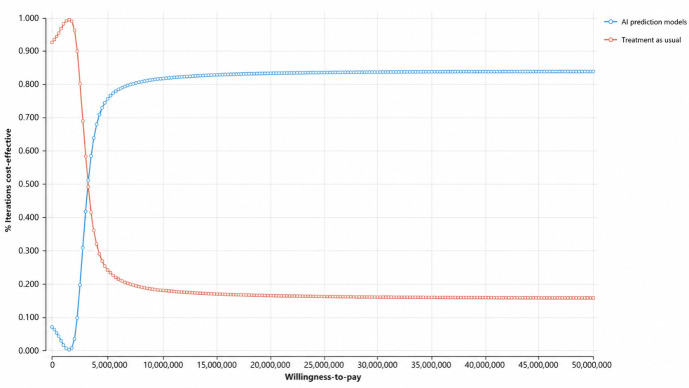
Cost-effectiveness acceptability curve for AI-assisted intensive care unit mortality prediction versus usual care.

The cost-effectiveness acceptability curve based on 100,000 probabilistic sensitivity analysis simulations is presented in Figure 2. The analysis used a lifetime Markov model for a hypothetical Korean adult general-population cohort, with AI monitoring activated upon ICU entry. At a WTP threshold of KRW 40 million (approximately US $29,600) per QALY, the probability that the AI-assisted strategy was cost-effective was approximately 83.94%.

In the AI cost threshold analysis, the AI-assisted strategy remained cost-effective until the AI implementation cost reached KRW 6,531,070 (approximately US $4840) per monitored admission, as detailed in Multimedia Appendix 13. At this point, the NMB of the AI-assisted strategy equaled that of usual care at a WTP threshold of KRW 40 million (approximately US $29,600) per QALY.

**Figure 3.**
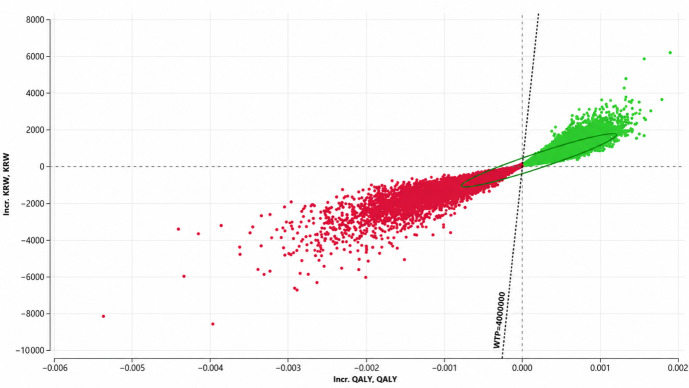
Incremental cost-effectiveness scatterplot for AI-assisted intensive care unit mortality prediction versus usual care. KRW: Korean won; QALY: quality-adjusted life year; WTP: willingness-to-pay.

The incremental cost-effectiveness scatterplot based on 100,000 probabilistic sensitivity analysis simulations is presented in Figure 3. Each point represents one simulation, with incremental QALYs shown on the *x*-axis and incremental costs in KRW shown on the *y*-axis. Most simulation results were located in the northeast quadrant, indicating slightly greater effectiveness and slightly higher costs for the AI-assisted strategy than for usual care.

### Scenario Analyses

Additional scenario analyses were conducted to assess structural and clinical uncertainty. Under shorter analytic horizons of 5 years and 10 years, and under more conservative mortality reduction assumptions of 10% and 5%, the AI-assisted strategy remained below the predefined WTP threshold. In supplementary post-ICU burden scenarios, progressively worse assumptions were applied to post-ICU utility, recurrent hospitalization or ICU-transition risk, and post-ICU mortality. The ICER remained stable, ranging from KRW 8,374,959 to KRW 8,387,761 (approximately US $6200‐$6210) per QALY, because these post-ICU burden assumptions affected both usual care and AI-assisted care in a similar direction. These findings are presented as supplementary stress-test results rather than primary base-case outcomes.

### One-Way Sensitivity Analysis

**Figure 4.**
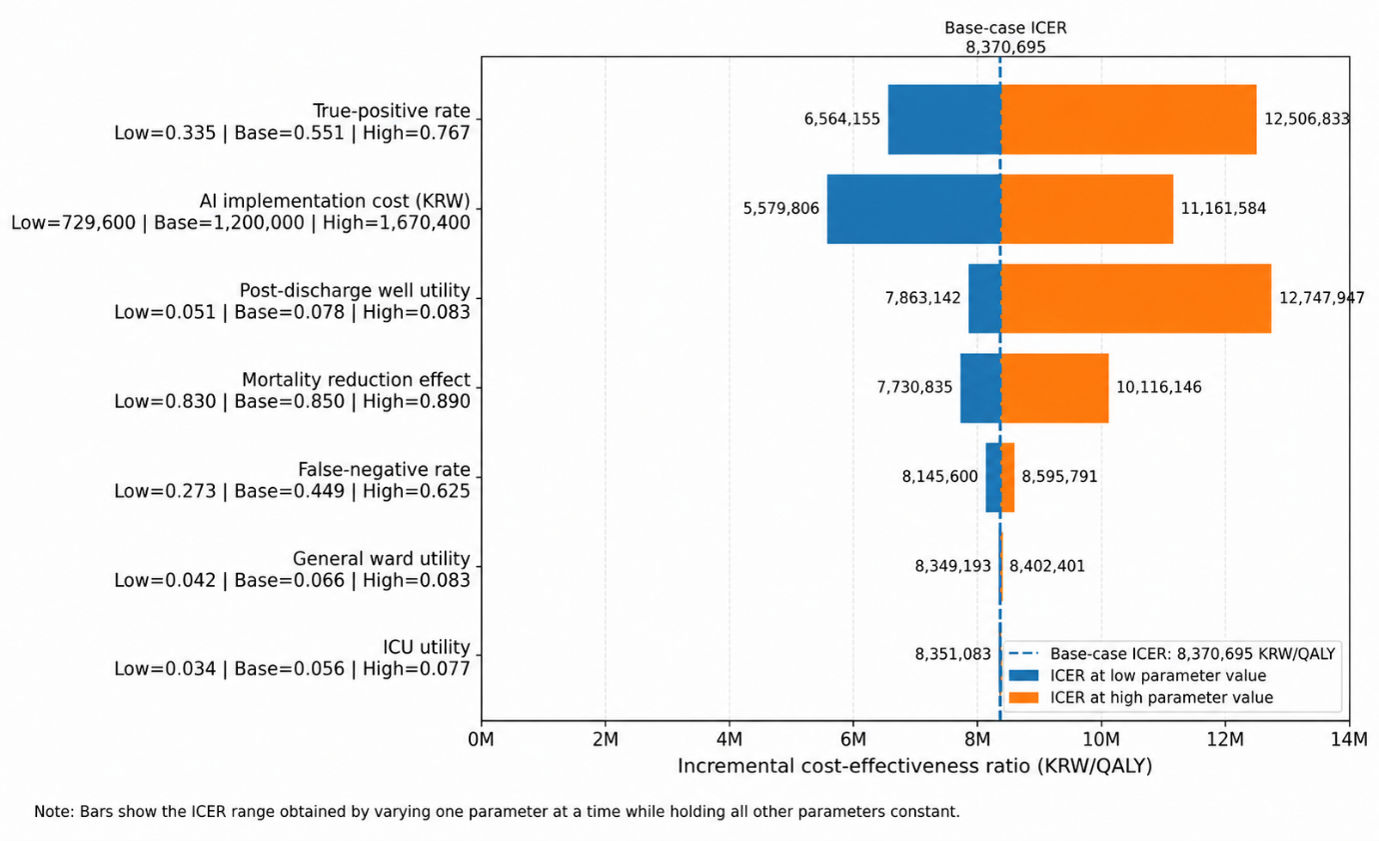
One-way sensitivity analysis of AI-assisted intensive care unit mortality prediction versus usual care. ICER: incremental cost-effectiveness ratio; ICU: intensive care unit; KRW: Korean won; QALY: quality-adjusted life year.

Figure 4 presents the 1-way sensitivity analysis tornado diagram for the ICER. This figure summarizes the effect of varying each key model parameter across its prespecified uncertainty range while holding all other parameters constant. The true-positive rate of the AI prediction model, AI implementation cost, and postdischarge well-state utility were the most influential parameters. The true-positive rate (Accuracy_TP) of the AI prediction model exerted the greatest influence on cost-effectiveness, with ICER values ranging from 6.56 to 12.51 million KRW (approximately US $4860‐$9270) per QALY, accounting for 36.7% of the overall variance. AI implementation cost was the second most influential factor. Across the tested range of KRW 729,600 to KRW 1,670,400 (approximately US $540‐$1237), the ICER varied from KRW 5.58 million to KRW 11.16 million (approximately US $4130‐$8270) per QALY and explained 32.4% of total variance. The utility value of the postdischarge well state ranked third, producing ICERs between KRW 7.86 million and KRW 12.75 million (approximately US $5820‐$9440) per QALY and accounting for 24.8% of variance. Across all alternative scenarios with reduced mortality effects (HR 0.90 and 0.95), the AI-based strategy remained cost-effective under the predefined WTP threshold (Multimedia Appendix 5).

### Real-World Analysis Using NHIS Data

Multimedia Appendix 7 summarizes descriptive statistics derived from NHIS-NSC data for 4 patient groups: GW inpatients, direct ICU admissions, ICU-to-GW transfers, and GW-to-ICU transfers. Across all observation periods (1, 3, and 6 months), ICU-related groups showed longer lengths of stay and higher medical costs than GW inpatients. Transition probabilities and cost parameters used in the Markov model were derived from these empirical analyses.

### Survival Outcomes by ICU Transition Pathway

Multimedia Appendices 8–10 present survival outcomes stratified by ICU transition pathway over 1-, 3-, and 6-month follow-up periods. Direct ICU admissions consistently showed higher mortality than GW inpatients across all time horizons (*P*<.001). Patients transferred from ICU to GW had lower mortality than those transferred from GW to ICU. Mortality increased with age across all patient groups, with the highest rates observed among patients aged ≥80 years. These findings provide contextual support for the age- and transition-dependent assumptions applied in the Markov model.

## Discussion

### Principal Findings

This study evaluated the potential cost-effectiveness of AI-assisted ICU mortality prediction using a decision-analytic Markov model informed by national claims data and published evidence. Under the base-case assumptions, the AI-assisted strategy showed an ICER of 8.37 million KRW (approximately US $6200) per QALY, which was below the commonly used Korean WTP threshold of 40 million KRW (approximately US $29,600) per QALY. However, these findings should be interpreted as modeled economic estimates rather than direct evidence that the AI system reduced ICU mortality in real-world patients. Probabilistic sensitivity analysis showed an 83.94% probability of cost-effectiveness at this threshold. The absolute incremental QALY gain was small, and 1-way sensitivity analysis identified the true-positive rate, AI implementation cost, and postdischarge well-state utility as the most influential parameters. These findings suggest that AI-assisted ICU mortality prediction may be economically favorable under the modeled assumptions, although its value depends strongly on diagnostic performance, implementation cost, and post-ICU recovery outcomes [27,28]. These results represent population-level lifetime expected values per member of the starting cohort and should not be interpreted as per-admission outcomes among patients already admitted to the ICU.

### Interpretation of Findings

AI prediction itself does not directly improve survival. Any potential benefit depends on whether risk information is translated into timely and appropriate clinical action, including earlier recognition, prioritization, escalation of monitoring, and intervention. Therefore, the mortality reduction parameter used in this study should be interpreted as a conditional system-level effect assumption rather than a direct causal effect of the algorithm alone. The true clinical impact of AI-assisted ICU mortality prediction may vary across institutions depending on workflow integration, clinician adherence, staffing, alert fatigue, and institutional readiness [29,30]. In particular, not all AI systems meet the diagnostic rigor required for CDSS, and inappropriate reliance on such tools may introduce risks such as automation bias [31]. High predictive accuracy alone does not guarantee improved patient outcomes [32]. The use of a general-population starting cohort allowed the model to capture the cumulative population-level consequences of both initial and recurrent ICU admissions over the lifetime horizon, rather than limiting the analysis to outcomes following a single-index ICU stay.

The small incremental QALY gain should also be interpreted cautiously. In critically ill ICU populations, large per-patient QALY gains are difficult to achieve because baseline mortality risk is high, short-term utility during acute illness is low, and many survivors experience persistent morbidity after discharge. Therefore, the favorable ICER observed in this study reflects the combination of modest modeled health gains and a small net incremental cost. These findings may be more relevant from a population-level efficiency perspective than as evidence of a large individual-level clinical benefit.

In practical implementation, the clinical value of AI alerts depends on whether they are embedded into a safe and actionable workflow [10]. False-positive alerts may increase workload and contribute to alert fatigue, whereas false-negative results may create safety concerns if clinicians place excessive trust in the absence of an alert [12]. Therefore, AI-based risk prediction should be implemented as a clinician-facing decision support tool with explicit response protocols, training, audit of alert burden, and periodic review of missed events. The findings of this study should be interpreted under this human-in-the-loop assumption rather than as evidence that AI alerts independently improve outcomes.

The post-ICU burden scenarios should be interpreted cautiously. These analyses were designed as supplementary stress tests rather than as alternative base-case assumptions. The 5%, 10%, 15%, and 20% changes represented progressively worse post-ICU trajectories, including reduced post-ICU quality of life, increased recurrent hospitalization or ICU-transition risk, and increased post-ICU mortality. The ICER remained relatively stable because these post-ICU burden assumptions affected both strategies in a similar direction. Consequently, the absolute total costs and QALYs changed, but the incremental difference between usual care and AI-assisted care changed only modestly.

### Comparison With Previous Literature

Previous research on AI prediction models in critical care has largely focused on algorithmic performance, such as area under the receiver operating characteristic curve (AUROC), calibration, or short-term mortality prediction [33,34]. For example, Chen et al [35] reported an AUROC of 0.865 using a random forest model, and Wu et al [36] proposed reinforcement learning–based treatment strategies without addressing cost-effectiveness. In contrast, this study extends this literature by linking AI-assisted risk prediction to costs, QALYs, and long-term economic outcomes.

The study also contributes to the emerging literature on economic evaluation of AI-based health care interventions. Internationally, economic evaluations of AI prediction models in ICU settings are increasing [37,38]. In the United States and Europe, ICU-focused AI prediction models have been evaluated in relation to resource allocation and potential health care cost implications [38,39]. The present findings are consistent with these global trends and provide locally relevant model-based evidence for considering AI-assisted prediction in the Korean critical care context [40].

### Policy and Implementation Implications

AI prediction models integrate vital signs and clinical data to identify high-risk patients early and may help clinicians prioritize patients who require closer monitoring or reassessment [5]. From a policy perspective, AI-assisted ICU mortality prediction may support more efficient allocation of clinical attention in resource-constrained critical care environments [41]. This may be particularly relevant in settings facing ICU capacity constraints, high clinician workload, and increasing demand for critical care services.

However, these potential system-level benefits require careful implementation. AI prediction models should not be understood as stand-alone solutions for improving ICU outcomes. Their value depends on clinical workflow integration, clinician response, alert governance, local implementation cost, and continuous monitoring of performance and safety. Such systems may contribute to care standardization and protocol adherence when appropriately implemented, but poorly governed implementation may increase workload, alert fatigue, or automation bias [42-44].

The socioeconomic implications of AI-assisted prediction should also be interpreted cautiously. Earlier recognition of high-risk ICU patients may reduce avoidable deterioration in some settings and may indirectly lessen family and societal burden [45,46]. In younger patients, survival after critical illness may have broader social and economic implications [47]. However, these broader implications were not directly measured in the present model and should be considered hypothesis-generating rather than definitive evidence of socioeconomic benefit.

The present findings should be interpreted within the Korean health care context. The WTP threshold, hospitalization costs, and AI implementation costs were based on Korean reimbursement structures and local operational assumptions. Therefore, direct transferability of the numerical ICER estimates to other settings should be approached cautiously. Nevertheless, the analytic framework used in this study may remain relevant internationally if local costs, thresholds, patient volumes, clinical workflows, and implementation conditions are appropriately adapted.

### Limitations

Several limitations should be noted. First, the mortality reduction parameter was not derived from a prospective trial directly evaluating the ICU mortality prediction system assessed in this study. Instead, the clinical benefit was modeled using assumptions informed by previous evidence on system-level critical care support. Therefore, the results should be interpreted as estimates of potential cost-effectiveness rather than proof that the AI system directly reduces ICU mortality. Second, the AI implementation cost of KRW 1,200,000 (approximately US $890) per monitored admission was a developer-provided, assumption-based estimate rather than a fully itemized hospital accounting value or a separately reimbursed AI-based patient monitoring fee. Although this cost varied in sensitivity analyses, actual implementation costs may differ across hospitals depending on licensing, infrastructure readiness, patient volume, staff training, and workflow integration. Third, the Markov model used a simplified hospital-centered structure. Although additional scenario analyses examined worse post-ICU utility, recurrent hospitalization or ICU transition, and post-ICU mortality assumptions, the model did not fully capture complex long-term pathways such as rehabilitation, long-term care, post-ICU complications, or repeated readmissions. Future studies using patient-level longitudinal data are needed to better estimate these downstream consequences. Finally, diagnostic performance inputs were derived from a published real-world implementation study and may vary across hospitals, patient populations, and implementation environments. The model also assumed that AI alerts would be appropriately interpreted and acted on by clinicians, but clinician response, alert fatigue, automation bias, and workflow disruption were not directly observed. Therefore, the small incremental QALY gain and favorable ICER should be interpreted as scenario-based economic evidence rather than definitive proof of individual-level clinical benefit. False-positive alerts and their downstream resource consequences were not separately quantified. Although specificity was reported, the model did not assign alert-specific costs for additional clinician review, bedside reassessment, diagnostic testing, or treatment. The aggregate implementation-cost assumption included routine workflow integration and operational support but did not explicitly capture marginal staff time per false-positive alert. The analysis may therefore underestimate costs in institutions where false-positive alerts result in substantial workload, alert fatigue, or additional testing.

### Conclusion

This population-level, model-based analysis suggests that an ICU-activated AI mortality prediction strategy may be cost-effective under the specified assumptions in Korea. These findings should not be interpreted as direct evidence that the AI system reduces mortality in real-world ICU patients. The results were favorable in the base-case and supplementary sensitivity analyses, with AI performance, implementation cost, and post-ICU utility identified as key determinants of cost-effectiveness. However, these findings should be interpreted as decision-analytic estimates rather than direct evidence of mortality reduction from AI use. Prospective real-world studies are needed to confirm the clinical effectiveness, implementation feasibility, and economic value of AI-assisted ICU mortality prediction.

## Supplementary material

10.2196/93466 Multimedia Appendix 1 Data sources and parameter derivation methods for the Markov model–based cost-utility analysis of AI-assisted intensive care unit mortality prediction in Korea.

10.2196/93466 Multimedia Appendix 2 Reference implementation study supporting diagnostic performance assumptions for the AI early warning system used in the intensive care unit mortality prediction cost-utility model.

10.2196/93466 Multimedia Appendix 3 AI implementation cost components, availability of itemized estimates, and allocation assumptions for the cost-utility analysis of AI-assisted intensive care unit mortality prediction.

10.2196/93466 Multimedia Appendix 4 Scenario analysis of alternative time horizons in the Markov model–based cost-utility analysis of AI-assisted intensive care unit mortality prediction among Korean adult intensive care unit patients.

10.2196/93466 Multimedia Appendix 5 Scenario analysis of alternative mortality reduction assumptions in the Markov model–based cost-utility analysis of AI-assisted intensive care unit mortality prediction among Korean adult intensive care unit patients.

10.2196/93466 Multimedia Appendix 6 Structural scenario analyses for post–intensive care unit pathway uncertainty in the Markov model–based cost-utility analysis of AI-assisted intensive care unit mortality prediction among Korean adult intensive care unit patients.

10.2196/93466 Multimedia Appendix 7 Descriptive characteristics and health care utilization outcomes by intensive care unit transition pathway and follow-up period among Korean adult inpatients.

10.2196/93466 Multimedia Appendix 8 One-month survival and mortality outcomes by intensive care unit transition pathway and age group among Korean adult inpatients.

10.2196/93466 Multimedia Appendix 9 Three-month survival and mortality outcomes by intensive care unit transition pathway and age group among Korean adult inpatients.

10.2196/93466 Multimedia Appendix 10 Six-month survival and mortality outcomes by intensive care unit transition pathway and age group among Korean adult inpatients.

10.2196/93466 Multimedia Appendix 11 Flowchart of National Health Insurance Service claims data cleaning and intensive care unit inpatient episode construction for the cost-utility analysis.

10.2196/93466 Multimedia Appendix 12 Assumed clinical workflow and safety considerations according to AI alert classification.

10.2196/93466 Multimedia Appendix 13 Threshold analysis of AI implementation cost using net monetary benefit at a willingness-to-pay threshold of Korean won 40 million per quality-adjusted life year.

10.2196/93466 Checklist 1 CHEERS-AI reporting checklist for the cost-utility analysis of AI-assisted intensive care unit mortality prediction.

## Abbreviations

AUROC: area under the receiver operating characteristic curve
CDSS: clinical decision support system
CEAC: cost-effectiveness acceptability curve
CHEERS-AI: Consolidated Health Economic Evaluation Reporting Standards for Interventions That Use Artificial Intelligence
DSA: deterministic sensitivity analysis
GW: general ward
HIRA: Health Insurance Review and Assessment Service
HR: hazard ratio
ICER: incremental cost-effectiveness ratio
ICU: intensive care unit
KRW: Korean won
ML: machine learning
NHIS: National Health Insurance Service
NHIS-NSC: National Health Insurance Service–National Sample Cohort
NMB: net monetary benefit
PSA: probabilistic sensitivity analysis
QALY: quality-adjusted life year
VC-MORS: VitalCare-Mortality Score
WTP: willingness-to-pay
