# Supplementary material for: Value of AI in Critical Care Using Real-World Evidence on Intensive Care Unit Mortality Prediction: Cost-Utility Analysis
Source: J Med Internet Res. 2026 Aug 18;28:e93466. doi: 10.2196/93466 (PMC13484949; doi:10.2196/93466)
Supplement: Supplementary file 1 [file jmir-v28-e93466-s001.docx]

This appendix summarizes the sources and derivation methods of model input parameters used in the economic evaluation.

**1. Diagnostic accuracy parameters**

Diagnostic accuracy parameters were derived from threshold-specific operating characteristics reported in the published real-world implementation study summarized in Supplementary Box S2. At the selected alert threshold of 39 points, sensitivity was 0.551 and specificity was 0.984. In the model, sensitivity was used as the true-positive classification probability, and the false-negative probability was calculated as 1 − sensitivity = 0.449.

**2. Mortality reduction parameter**

The mortality reduction parameter (hazard ratio = 0.85) was informed by a nationwide Korean cohort study evaluating intensivist coverage (Oh & Song, 2023). This value was used as a proxy estimate for potential system-level benefit associated with earlier risk recognition and timely clinical response.

**3. Healthcare utilization parameters**

Because the model used monthly cycles, annual utilization statistics were converted to monthly transition probabilities.

- Admission rates were derived from 2023 population and healthcare utilization statistics published by the Ministry of the Interior and Safety, Republic of Korea.
- Discharge rates were derived from the Health Insurance Review & Assessment Service (HIRA) Healthcare Utilization Statistics.
- Patients in the post-discharge well state were assumed to remain alive in that state unless age-specific background mortality occurred.

**4. Transition probabilities and cost inputs**

Clinical transition probabilities were estimated using the NHIS National Sample Cohort (2008–2019), including age-stratified transitions among ICU admission, ward hospitalization, discharge, and death.

Hospitalization costs (ICU and GW) were also estimated from NHIS claims data. AI implementation costs were modeled separately using scenario-based estimates incorporating licensing, infrastructure, maintenance, and operational deployment assumptions.

**5. Utility values**

Health utility values were obtained from previously published studies and represented quality-of-life weights associated with ICU admission, ward hospitalization, and post-discharge survival states.

**Note**. This box summarizes the sources and derivation methods for diagnostic accuracy, mortality reduction, healthcare utilization, transition probabilities, costs, and utility values used in the economic model. The model evaluated AI-assisted ICU mortality prediction versus usual care among Korean adults aged 19 years or older admitted to intensive care units.
