# Supplementary material for: Value of AI in Critical Care Using Real-World Evidence on Intensive Care Unit Mortality Prediction: Cost-Utility Analysis
Source: J Med Internet Res. 2026 Aug 18;28:e93466. doi: 10.2196/93466 (PMC13484949; doi:10.2196/93466)
Supplement: Supplementary file 2 [file jmir-v28-e93466-s002.docx]

- Title: Deep Learning–Based Early Warning Systems in Hospitalized Patients at Risk of Code Blue Events and Length of Stay: Retrospective Real-World Implementation Study
- Journal: JMIR Medical Informatics (2025)
- DOI: 10.2196/72232
- Study Setting: Presbyterian Medical Center, Republic of Korea
- Study Design: Retrospective real-world implementation study (pre-post design)
- Study Population:
  - Hospitalized patients admitted to GWs and intensive care units between December 2022 and May 2024.
  - Data from March 2023 were excluded as a system stabilization period following implementation.
  - Total of 30,785 hospitalizations in the study cohort.
- Results of Study
  - Model Performance

| **Score** | **AUROC (bootstrap 95% CI)** | **AUPRC (bootstrap 95% CI)** |
| --- | --- | --- |
| **GWs** | | |
| VC-MAES | 0.865 (0.857‐0.873) | 0.088 (0.080‐0.098) |
| NEWS | 0.804 (0.797‐0.811) | 0.018 (0.015‐0.021) |
| MEWS | 0.772 (0.764-0.780) | 0.020 (0.015‐0.025) |
| **Intensive care units** | | |
| VC-MORS | 0.937 (0.931‐0.944) | 0.098 (0.087‐0.109) |
| SOFA | 0.754 (0.742-0.765) | 0.011 (0.010‐0.012) |
| APACHE II | 0.814 (0.787‐0.840) | 0.259 (0.215‐0.307) |

- Detailed characteristic value corresponding to a cut-off of 39 points,
  - Sensitivity: 0.551
    Specificity: 0.984

**Resource**: Kim, et al. Deep Learning–Based Early Warning Systems in Hospitalized Patients at Risk of Code Blue Events and Length of Stay: Retrospective Real-World Implementation Study (2025).

**Note**. This box summarizes the published real-world implementation study used to inform diagnostic performance inputs for the AI early warning system. The source implementation study included hospitalized patients admitted to general wards and intensive care units in the Republic of Korea; however, the present economic evaluation used only the ICU-specific VC-MORS performance estimates. Threshold-specific sensitivity and specificity at a cut-off score of 39 points for VC-MORS in ICU patients were used to inform the diagnostic performance assumptions in the economic model.
