# Supplementary material for: Value of AI in Critical Care Using Real-World Evidence on Intensive Care Unit Mortality Prediction: Cost-Utility Analysis
Source: J Med Internet Res. 2026 Aug 18;28:e93466. doi: 10.2196/93466 (PMC13484949; doi:10.2196/93466)
Supplement: Supplementary file 3 [file jmir-v28-e93466-s003.docx]

| **Cost component** | **Description** | **Included in aggregate AI cost?** | **Separate itemized estimate available?** | **How handled in the model** |
| --- | --- | --- | --- | --- |
| Software licensing | License or subscription cost for use of the AI early warning system | Yes | Not separately available | Included in the aggregate allocated AI implementation cost per monitored admission |
| Server and infrastructure | Server, data interface, storage, monitoring dashboard, and technical deployment infrastructure | Yes | Not separately available | Included in the aggregate allocated AI implementation cost |
| Maintenance and technical support | Software updates, troubleshooting, technical monitoring, and vendor support | Yes | Not separately available | Included in the aggregate allocated AI implementation cost |
| Staff training | Initial and recurrent training for clinicians and users regarding alert interpretation and workflow response | Yes, implementation-dependent | Not separately available | Reflected as part of implementation-related cost assumptions |
| Workflow integration | Local alert protocol development, dashboard configuration, and integration into clinical workflow | Yes, implementation-dependent | Not separately available | Reflected as part of implementation-related cost assumptions |
| Operational management | Ongoing monitoring of alert burden, false-positive alerts, missed cases, user feedback, and local performance review | Yes, implementation-dependent | Not separately available | Reflected as part of implementation-related cost assumptions |
| Patient volume | Annual monitored ICU admissions used to allocate system-level cost to patient-level model input | Required for allocation | Not directly available as a fixed universal value | Addressed through per-admission costing and sensitivity/threshold analyses |
| Base-case allocated AI cost | Average AI implementation cost per monitored admission | Yes | Aggregate estimate available | KRW 1,200,000 per monitored admission |
| Uncertainty about range of AI cost | Plausible lower and upper values for AI implementation cost | Yes | Aggregate estimate available | KRW 729,600–1,670,400 per monitored admission; varied in deterministic and probabilistic sensitivity analyses |
| Cost-effectiveness threshold for AI cost | AI cost level at which the ICER equals KRW 40 million/QALY | Not a vendor price; model-derived threshold | Not applicable | Estimated through threshold analysis and reported in the Results section and Supplementary Figure S2 |

**Note.** The AI implementation cost was modeled as an aggregate allocated cost per monitored admission rather than as separately itemized component-level prices. Component-level commercial estimates for licensing, infrastructure, maintenance, training, workflow integration, and operational management were not separately available. Therefore, arbitrary component-level prices were not imputed. Instead, these components were transparently described as included in the aggregate cost parameter, and uncertainty was addressed through deterministic sensitivity analysis, probabilistic sensitivity analysis, and threshold analysis. Actual implementation costs may vary by hospital size, patient volume, licensing arrangement, infrastructure readiness, staffing requirements, and local workflow integration. Costs are presented in 2023 KRW; approximate USD equivalents can be calculated using KRW 1,350=USD 1.
