# Supplementary material for: Value of AI in Critical Care Using Real-World Evidence on Intensive Care Unit Mortality Prediction: Cost-Utility Analysis
Source: J Med Internet Res. 2026 Aug 18;28:e93466. doi: 10.2196/93466 (PMC13484949; doi:10.2196/93466)
Supplement: Supplementary file 4 [file jmir-v28-e93466-s004.docx]

| **Scenario** | **Strategy** | **Total Cost (KRW)** | **Incremental Cost (KRW)** | **QALYs** | **Incremental QALYs** | **ICER (KRW/QALY)** | **NMB (KRW)*** |
| --- | --- | --- | --- | --- | --- | --- | --- |
| **5-year horizon** | Treatment as usual | 1,823,710 | – | 13.7467725 | – | – | 548,047,189 |
|  | AI Prediction Model | 1,824,116 | 406 | 13.7467950 | 0.0000225 | 18,017,023 | 548,047,684 |
| **10-year horizon** | Treatment as usual | 2,014,512 | – | 15.1786317 | – | – | 605,130,757 |
|  | AI Prediction Model | 2,014,962 | 449 | 15.1786606 | 0.0000289 | 15,546,380 | 605,131,464 |

**Note**. This table presents scenario analyses using 5-year and 10-year time horizons compared with the lifetime base-case model. The analysis compared AI-assisted ICU mortality prediction with usual care from the Korean payer perspective. Net monetary benefit was calculated using a willingness-to-pay threshold of KRW 40 million per QALY. Costs are presented in 2023 KRW; approximate USD equivalents can be calculated using KRW 1,350=USD 1.
