# Supplementary material for: Value of AI in Critical Care Using Real-World Evidence on Intensive Care Unit Mortality Prediction: Cost-Utility Analysis
Source: J Med Internet Res. 2026 Aug 18;28:e93466. doi: 10.2196/93466 (PMC13484949; doi:10.2196/93466)
Supplement: Supplementary file 5 [file jmir-v28-e93466-s005.docx]

| **Scenario** | **Strategy** | **Total Cost (KRW)** | **Incremental Cost (KRW)** | **QALYs** | **Incremental QALYs** | **ICER (KRW/QALY)** | **NMB (KRW)*** |
| --- | --- | --- | --- | --- | --- | --- | --- |
| **10% mortality reduction** | Treatment as usual | 2,746,228 | – | 20.669723 | – | – | 824,042,703 |
|  | AI Prediction Model | 2,746,822 | 593 | 20.669779 | 0.000055 | 10,693,392 | 824,044,329 |
| **5% mortality reduction** | Treatment as usual | 2,746,228 | – | 20.669723 | – | – | 824,042,703 |
|  | AI Prediction Model | 2,746,796 | 568 | 20.669761 | 0.000037 | 15,230,936 | 824,043,627 |

**Note**. This table presents scenario analyses using more conservative mortality reduction assumptions than the base-case model. The analysis compared AI-assisted ICU mortality prediction with usual care among Korean adult ICU patients from the Korean payer perspective. Net monetary benefit was calculated using a willingness-to-pay threshold of KRW 40 million per QALY. Costs are presented in 2023 KRW; approximate USD equivalents can be calculated using KRW 1,350=USD 1.
