# Supplementary material for: Value of AI in Critical Care Using Real-World Evidence on Intensive Care Unit Mortality Prediction: Cost-Utility Analysis
Source: J Med Internet Res. 2026 Aug 18;28:e93466. doi: 10.2196/93466 (PMC13484949; doi:10.2196/93466)
Supplement: Supplementary file 6 [file jmir-v28-e93466-s006.docx]

| Scenario | Structural uncertainty addressed | Strategy | Total cost (KRW) | Incremental cost (KRW) | QALYs | Incremental QALYs | ICER (KRW/QALY) | NMB (KRW) |
| --- | --- | --- | --- | --- | --- | --- | --- | --- |
| Mild post-discharge burden scenario, 5% variation | Conservative variation reflecting omitted post-ICU burden | Treatment as usual | 2,746,228 | – | 20.669721 | – | – | 824,042,622 |
|  |  | AI Prediction Model | 2,746,847 | 619 | 20.669795 | 0.0000739 | 8,374,959 | 824,044,958 |
| Moderate post-discharge burden scenario, 10% variation | Conservative variation reflecting omitted post-ICU burden | Treatment as usual | 2,746,228 | – | 20.669719 | – | – | 824,042,541 |
|  |  | AI Prediction Model | 2,746,847 | 619 | 20.669793 | 0.0000738 | 8,379,224 | 824,044,876 |
| Severe post-discharge burden scenario, 15% variation | Conservative variation reflecting omitted post-ICU burden | Treatment as usual | 2,746,228 | – | 20.669717 | – | – | 824,042,460 |
|  |  | AI Prediction Model | 2,746,846 | 619 | 20.669791 | 0.0000738 | 8,383,491 | 824,044,793 |
| Combined conservative post-discharge burden scenario, 20% variation | Combined conservative assumption for omitted post-ICU burden | Treatment as usual | 2,746,227 | – | 20.669715 | – | – | 824,042,378 |
|  |  | AI Prediction Model | 2,746,846 | 619 | 20.669789 | 0.0000738 | 8,387,761 | 824,044,710 |

**Note**. These analyses were supplementary stress-test scenarios and were not interpreted as base-case results. Post-ICU utility was reduced to 95%, 90%, 85%, and 80% of the base-case value. Recurrent hospitalization or ICU-transition risk and post-ICU mortality risk were increased by 5%, 10%, 15%, and 20%, respectively. These changes were intended to reflect progressively worse recurrent post-ICU event burden. Because these assumptions were applied similarly to both strategies, incremental costs and incremental QALYs changed only modestly, resulting in relatively stable ICERs. Costs are presented in 2023 KRW; approximate USD equivalents can be calculated using KRW 1,350=USD 1.
