# Supplementary material for: Value of AI in Critical Care Using Real-World Evidence on Intensive Care Unit Mortality Prediction: Cost-Utility Analysis
Source: J Med Internet Res. 2026 Aug 18;28:e93466. doi: 10.2196/93466 (PMC13484949; doi:10.2196/93466)
Supplement: Supplementary file 7 [file jmir-v28-e93466-s007.docx]

(Unit: 10,000 persons; years; days; 10,000KRW; N)

| **Group** |  |  | **<=1-Month** | | | | | | **<=3-Month** | | | | | | **<=6-Month** | | | | | |
| --- | --- | --- | --- | --- | --- | --- | --- | --- | --- | --- | --- | --- | --- | --- | --- | --- | --- | --- | --- | --- |
|  | **Variable** | | **Num** | **Mean** | **SD** | **Median** | **Q1** | **Q3** | **Num** | **Mean** | **SD** | **Median** | **Q1** | **Q3** | **Num** | **Mean** | **SD** | **Median** | **Q1** | **Q3** |
| GW | Age | | 10,109 | 55.7 | 17.6 | 57 | 43 | 69 | 10,120 | 56.2 | 17.8 | 58 | 43 | 70 | 10,122 | 56.7 | 17.8 | 58 | 44 | 71 |
|  | LoS | |  | 1.6 | 2.1 | 1 | 1 | 1 |  | 1.7 | 3.5 | 1 | 1 | 1 |  | 1.7 | 4.4 | 1 | 1 | 1 |
|  | Cost | |  | 12 | 59 | 2 | 1 | 5 |  | 14 | 83 | 2 | 1 | 5 |  | 13 | 88 | 2 | 1 | 5 |
|  | Surgeries | |  | 2 | 54 | 0 | 0 | 0 |  | 2 | 81 | 0 | 0 | 0 |  | 2 | 315 | 0 | 0 | 0 |
| ICU | Age | | 1.1 | 70.8 | 14.1 | 74 | 62 | 81 | 1.6 | 71.5 | 13.7 | 75 | 63 | 82 | 1.7 | 71.7 | 13.6 | 75 | 64 | 82 |
|  | LoS | |  | 12.3 | 7.7 | 11 | 6 | 18 |  | 23.5 | 18.8 | 18 | 9 | 33 |  | 30.9 | 30.8 | 21 | 10 | 40 |
|  | Cost | |  | 888 | 920 | 614 | 315 | 1130 |  | 1,331 | 1,466 | 891 | 442 | 1,674 |  | 1,532 | 1,797 | 982 | 481 | 1,893 |
|  | Surgeries | |  | 5 | 36 | 9 | 9 | 9 |  | 6 | 54 | 9 | 9 | 9 |  | 7 | 72 | 9 | 9 | 9 |
| ICU > GW | Age | | 4.4 | 69 | 14.9 | 71 | 60 | 81 | 5.0 | 69.6 | 14.5 | 72 | 61 | 80 | 5.1 | 69.2 | 14.3 | 72 | 60 | 80 |
|  | LoS | |  | 10.2 | 5.8 | 9 | 6 | 12 |  | 14.8 | 11 | 12 | 7 | 19 |  | 17.6 | 15.1 | 13 | 8 | 23 |
|  | Cost | |  | 726 | 733 | 494 | 252 | 944 |  | 823 | 780 | 607 | 313 | 1,060 |  | 927 | 982 | 682 | 348 | 1,167 |
|  | Surgeries | |  | 5 | 18 | 9 | 9 | 9 |  | 5 | 36 | 9 | 9 | 9 |  | 5 | 36 | 9 | 9 | 9 |
| GW > ICU | Age | | 5.4 | 71.6 | 13.5 | 75 | 64 | 81 | 5.4 | 71.4 | 13.8 | 74 | 63 | 81 | 5.4 | 71 | 14 | 74 | 63 | 81 |
|  | LoS | |  | 2.2 | 2.9 | 1 | 1 | 2 |  | 2.9 | 5.9 | 1 | 1 | 2 |  | 3 | 7 | 1 | 1 | 2 |
|  | Cost | |  | 26 | 80 | 2 | 1 | 11 |  | 41 | 149 | 2 | 1 | 13 |  | 41 | 160 | 2 | 1 | 12 |
|  | Surgeries | |  | 1 | 18 | 0 | 0 | 0 |  | 2 | 27 | 0 | 0 | 0 |  | 2 | 27 | 0 | 0 | 0 |

**Note**. This table summarizes age, length of stay, medical costs, and number of surgeries according to hospitalization pathway and follow-up period. Groups included general ward inpatients, direct ICU admissions, ICU-to-general ward transfers, and general ward-to-ICU transfers. Cost is presented in units of KRW × 10⁴.
