# Supplementary material for: Value of AI in Critical Care Using Real-World Evidence on Intensive Care Unit Mortality Prediction: Cost-Utility Analysis
Source: J Med Internet Res. 2026 Aug 18;28:e93466. doi: 10.2196/93466 (PMC13484949; doi:10.2196/93466)
Supplement: Supplementary file 8 [file jmir-v28-e93466-s008.docx]

|  | Category | <=1-Month | | | | | | | | | | |
| --- | --- | --- | --- | --- | --- | --- | --- | --- | --- | --- | --- | --- |
|  |  | GW | | ICU | | ICU > GW | | GW > ICU | | Total | | p-value |
|  |  | N | % | N | % | N | % | N | % | N | % |  |
| **Total** | | 101,098,480 |  | 11,935 |  | 44,159 |  | 54,604 |  | 101,209,178 |  | <0.001 |
|  | Survival | 92,811,587 | 91.80 | 3,392 | 28.42 | 29,758 | 67.39 | 30,710 | 56.24 | 92,875,447 | 91.77 |  |
|  | Death | 8,286,893 | 8.20 | 8,543 | 71.58 | 14,401 | 32.61 | 23,894 | 43.76 | 8,333,731 | 8.23 |  |
| **Age(19-29)** | | 10,239,727 |  | 122 |  | 1,218 |  | 1,292 |  | 10,242,359 |  | <0.001 |
|  | Survival | 10,191,328 | 99.53 | 73 | 59.84 | 1,140 | 93.60 | 1,150 | 89.01 | 10,193,691 | 99.52 |  |
|  | Death | 48,399 | 0.47 | 49 | 40.16 | 78 | 6.40 | 142 | 10.99 | 48,668 | 0.48 |  |
| **Age(30-39)** | | 12,903,375 |  | 281 |  | 2,034 |  | 2,187 |  | 12,907,877 |  | <0.001 |
|  | Survival | 12,792,510 | 99.14 | 130 | 46.26 | 1,817 | 89.33 | 1,807 | 82.62 | 12,796,264 | 99.14 |  |
|  | Death | 110,865 | 0.86 | 151 | 53.74 | 217 | 10.67 | 380 | 17.38 | 111,613 | 0.86 |  |
| **Age(40-49)** | | 16,025,497 |  | 789 |  | 4,718 |  | 5,184 |  | 16,036,188 |  | <0.001 |
|  | Survival | 15,738,333 | 98.21 | 328 | 41.57 | 3,943 | 83.57 | 3,912 | 75.46 | 15,746,516 | 98.19 |  |
|  | Death | 287,164 | 1.79 | 461 | 58.43 | 775 | 16.43 | 1,272 | 24.54 | 289,672 | 1.81 |  |
| **Age(50-59)** | | 20,909,716 |  | 1,463 |  | 8,438 |  | 9,505 |  | 20,929,122 |  | <0.001 |
|  | Survival | 20,213,131 | 96.67 | 576 | 39.37 | 6,814 | 80.75 | 6,899 | 72.58 | 20,227,420 | 96.65 |  |
|  | Death | 696,585 | 3.33 | 887 | 60.63 | 1,624 | 19.25 | 2,606 | 27.42 | 701,702 | 3.35 |  |
| **Age(60-69)** | | 19,135,546 |  | 1,979 |  | 10,258 |  | 11,969 |  | 19,159,752 |  | <0.001 |
|  | Survival | 17,653,831 | 92.26 | 591 | 29.86 | 7,508 | 73.19 | 7,726 | 64.55 | 17,669,656 | 92.22 |  |
|  | Death | 1,481,715 | 7.74 | 1,388 | 70.14 | 2,750 | 26.81 | 4,243 | 35.45 | 1,490,096 | 7.78 |  |
| **Age(70-79)** | | 15,978,894 |  | 3,522 |  | 11,124 |  | 14,509 |  | 16,008,049 |  | <0.001 |
|  | Survival | 12,707,702 | 79.53 | 880 | 24.99 | 6,158 | 55.36 | 6,519 | 44.93 | 12,721,259 | 79.47 |  |
|  | Death | 3,271,192 | 20.47 | 2,642 | 75.01 | 4,966 | 44.64 | 7,990 | 55.07 | 3,286,790 | 20.53 |  |
| **Age(>=80)** | | 5,905,725 |  | 3,779 |  | 6,369 |  | 9,958 |  | 5,925,831 |  | <0.001 |
|  | Survival | 3,514,752 | 59.51 | 814 | 21.54 | 2,378 | 37.34 | 2,697 | 27.08 | 3,520,641 | 59.41 |  |
|  | Death | 2,390,973 | 40.49 | 2,965 | 78.46 | 3,991 | 62.66 | 7,261 | 72.92 | 2,405,190 | 40.59 |  |

**Note**. This table presents one-month survival and mortality outcomes stratified by hospitalization pathway and age group. Groups included general ward inpatients, direct ICU admissions, ICU-to-general ward transfers, and general ward-to-ICU transfers. P values compare survival and mortality distributions across groups.
