# Supplementary material for: Value of AI in Critical Care Using Real-World Evidence on Intensive Care Unit Mortality Prediction: Cost-Utility Analysis
Source: J Med Internet Res. 2026 Aug 18;28:e93466. doi: 10.2196/93466 (PMC13484949; doi:10.2196/93466)
Supplement: Supplementary file 9 [file jmir-v28-e93466-s009.docx]

| Category | | <=3-Month | | | | | | | | | | |
| --- | --- | --- | --- | --- | --- | --- | --- | --- | --- | --- | --- | --- |
|  |  | GW | | ICU | | ICU > GW | | GW > ICU | | Total | | p-value |
|  |  | N | % | N | % | N | % | N | % | N | % |  |
| **Total** | | 101,204,517 |  | 16,613 |  | 50,605 |  | 54,899 |  | 101,326,634 |  | <0.001 |
|  | Survival | 92,884,324 | 91.78 | 3,999 | 24.07 | 33,127 | 65.46 | 30,782 | 56.07 | 92,952,232 | 91.74 |  |
|  | Death | 8,320,193 | 8.22 | 12,614 | 75.93 | 17,478 | 34.54 | 24,117 | 43.93 | 8,374,402 | 8.26 |  |
| **Age(19-29)** | | 10,243,392 |  | 155 |  | 1,381 |  | 1,295 |  | 10,246,223 |  | <0.001 |
|  | Survival | 10,194,808 | 99.53 | 89 | 57.42 | 1,272 | 92.11 | 1,150 | 88.80 | 10,197,319 | 99.52 |  |
|  | Death | 48,584 | 0.47 | 66 | 42.58 | 109 | 7.89 | 145 | 11.20 | 48,904 | 0.48 |  |
| **Age(30-39)** | | 12,909,589 |  | 371 |  | 2,286 |  | 2,199 |  | 12,914,445 |  | <0.001 |
|  | Survival | 12,798,055 | 99.14 | 165 | 44.47 | 2,016 | 88.19 | 1,812 | 82.40 | 12,802,048 | 99.13 |  |
|  | Death | 111,534 | 0.86 | 206 | 55.53 | 270 | 11.81 | 387 | 17.60 | 112,397 | 0.87 |  |
| **Age(40-49)** | | 16,038,163 |  | 1,057 |  | 5,301 |  | 5,223 |  | 16,049,744 |  | <0.001 |
|  | Survival | 15,748,736 | 98.20 | 409 | 38.69 | 4,359 | 82.23 | 3,924 | 75.13 | 15,757,428 | 98.18 |  |
|  | Death | 289,427 | 1.80 | 648 | 61.31 | 942 | 17.77 | 1,299 | 24.87 | 292,316 | 1.82 |  |
| **Age(50-59)** | | 20,930,996 |  | 2,008 |  | 9,580 |  | 9,567 |  | 20,952,151 |  | <0.001 |
|  | Survival | 20,230,236 | 96.65 | 707 | 35.21 | 7,563 | 78.95 | 6,917 | 72.30 | 20,245,423 | 96.63 |  |
|  | Death | 700,760 | 3.35 | 1,301 | 64.79 | 2,017 | 21.05 | 2,650 | 27.70 | 706,728 | 3.37 |  |
| **Age(60-69)** | | 19,157,626 |  | 2,842 |  | 11,639 |  | 12,027 |  | 19,184,134 |  | <0.001 |
|  | Survival | 17,670,013 | 92.23 | 708 | 24.91 | 8,300 | 71.31 | 7,744 | 64.39 | 17,686,765 | 92.19 |  |
|  | Death | 1,487,613 | 7.77 | 2,134 | 75.09 | 3,339 | 28.69 | 4,283 | 35.61 | 1,497,369 | 7.81 |  |
| **Age(70-79)** | | 16,004,733 |  | 5,021 |  | 13,070 |  | 14,571 |  | 16,037,395 |  | <0.001 |
|  | Survival | 12,722,596 | 79.49 | 1,015 | 20.22 | 6,938 | 53.08 | 6,531 | 44.82 | 12,737,080 | 79.42 |  |
|  | Death | 3,282,137 | 20.51 | 4,006 | 79.78 | 6,132 | 46.92 | 8,040 | 55.18 | 3,300,315 | 20.58 |  |
| **Age(>=80)** | | 5,920,018 |  | 5,159 |  | 7,348 |  | 10,017 |  | 5,942,542 | 100.00 | <0.001 |
|  | Survival | 3,519,880 | 59.46 | 906 | 17.56 | 2,679 | 36.46 | 2,704 | 26.99 | 3,526,169 | 59.34 |  |
|  | Death | 2,400,138 | 40.54 | 4,253 | 82.44 | 4,669 | 63.54 | 7,313 | 73.01 | 2,416,373 | 40.66 |  |

**Note**. This table presents three-month survival and mortality outcomes stratified by hospitalization pathway and age group. Groups included general ward inpatients, direct ICU admissions, ICU-to-general ward transfers, and general ward-to-ICU transfers. P values compare survival and mortality distributions across groups.
