# Supplementary material for: Value of AI in Critical Care Using Real-World Evidence on Intensive Care Unit Mortality Prediction: Cost-Utility Analysis
Source: J Med Internet Res. 2026 Aug 18;28:e93466. doi: 10.2196/93466 (PMC13484949; doi:10.2196/93466)
Supplement: Supplementary file 10 [file jmir-v28-e93466-s010.docx]

| Category | | <=6-Month | | | | | | | | | | |
| --- | --- | --- | --- | --- | --- | --- | --- | --- | --- | --- | --- | --- |
|  |  | GW | | ICU | | ICU > GW | | GW > ICU | | Total | | p-value |
|  |  | N | % | N | % | N | % | N | % | N | % |  |
| **Total** | | 101,221,809 |  | 17,787 |  | 51,116 |  | 54,940 |  | 101,345,652 |  | <0.001 |
|  | Survival | 92,894,259 | 91.77 | 4,124 | 23.19 | 33,358 | 65.26 | 30,791 | 56.04 | 92,962,532 | 91.73 |  |
|  | Death | 8,327,550 | 8.23 | 13,663 | 76.81 | 17,758 | 34.74 | 24,149 | 43.96 | 8,383,120 | 8.27 |  |
| **Age(19-29)** | | 10,243,880 |  | 169 |  | 1,397 |  | 1,297 |  | 10,246,743 |  | <0.001 |
|  | Survival | 10,195,249 | 99.53 | 91 | 53.85 | 1,286 | 92.05 | 1,152 | 88.82 | 10,197,778 | 99.52 |  |
|  | Death | 48,631 | 0.47 | 78 | 46.15 | 111 | 7.95 | 145 | 11.18 | 48,965 | 0.48 |  |
| **Age(30-39)** | | 12,910,677 |  | 397 |  | 2,309 |  | 2,200 |  | 12,915,583 |  | <0.001 |
|  | Survival | 12,798,994 | 99.13 | 170 | 42.82 | 2,033 | 88.05 | 1,813 | 82.41 | 12,803,010 | 99.13 |  |
|  | Death | 111,683 | 0.87 | 227 | 57.18 | 276 | 11.95 | 387 | 17.59 | 112,573 | 0.87 |  |
| **Age(40-49)** | | 16,040,512 |  | 1,118 |  | 5,360 |  | 5,232 |  | 16,052,222 |  | <0.001 |
|  | Survival | 15,750,561 | 98.19 | 423 | 37.84 | 4,401 | 82.11 | 3,929 | 75.10 | 15,759,314 | 98.18 |  |
|  | Death | 289,951 | 1.81 | 695 | 62.16 | 959 | 17.89 | 1,303 | 24.90 | 292,908 | 1.82 |  |
| **Age(50-59)** | | 20,934,503 |  | 2,129 |  | 9,660 |  | 9,571 |  | 20,955,863 |  | <0.001 |
|  | Survival | 20,232,798 | 96.65 | 728 | 34.19 | 7,600 | 78.67 | 6,918 | 72.28 | 20,248,044 | 96.62 |  |
|  | Death | 701,705 | 3.35 | 1,401 | 65.81 | 2,060 | 21.33 | 2,653 | 27.72 | 707,819 | 3.38 |  |
| **Age(60-69)** | | 19,160,461 |  | 3,047 |  | 11,732 |  | 12,037 |  | 19,187,277 |  | <0.001 |
|  | Survival | 17,671,719 | 92.23 | 730 | 23.96 | 8,347 | 71.15 | 7,744 | 64.33 | 17,688,540 | 92.19 |  |
|  | Death | 1,488,742 | 7.77 | 2,317 | 76.04 | 3,385 | 28.85 | 4,293 | 35.67 | 1,498,737 | 7.81 |  |
| **Age(70-79)** | | 16,008,284 |  | 5,409 |  | 13,228 |  | 14,578 |  | 16,041,499 | 100.00 | <0.001 |
|  | Survival | 12,724,092 | 79.48 | 1,045 | 19.32 | 6,986 | 52.81 | 6,531 | 44.80 | 12,738,654 | 79.41 |  |
|  | Death | 3,284,192 | 20.52 | 4,364 | 80.68 | 6,242 | 47.19 | 8,047 | 55.20 | 3,302,845 | 20.59 |  |
| **Age(>=80)** | | 5,923,492 |  | 5,518 |  | 7,430 |  | 10,025 |  | 5,946,465 |  | <0.001 |
|  | Survival | 3,520,846 | 59.44 | 937 | 16.98 | 2,705 | 36.41 | 2,704 | 26.97 | 3,527,192 | 59.32 |  |
|  | Death | 2,402,646 | 40.56 | 4,581 | 83.02 | 4,725 | 63.59 | 7,321 | 73.03 | 2,419,273 | 40.68 |  |

**Note**. This table presents six-month survival and mortality outcomes stratified by hospitalization pathway and age group. Groups included general ward inpatients, direct ICU admissions, ICU-to-general ward transfers, and general ward-to-ICU transfers. P values compare survival and mortality distributions across groups.
