# Supplementary material for: Value of AI in Critical Care Using Real-World Evidence on Intensive Care Unit Mortality Prediction: Cost-Utility Analysis
Source: J Med Internet Res. 2026 Aug 18;28:e93466. doi: 10.2196/93466 (PMC13484949; doi:10.2196/93466)
Supplement: Supplementary file 11 [file jmir-v28-e93466-s011.docx]

**Note**. This figure shows the claims data screening, duplicate removal, washout, and ICU episode construction process used to identify inpatient episodes for estimating transition probabilities, healthcare utilization patterns, and age-stratified cost inputs in the Markov model.
