# Supplementary material for: Value of AI in Critical Care Using Real-World Evidence on Intensive Care Unit Mortality Prediction: Cost-Utility Analysis
Source: J Med Internet Res. 2026 Aug 18;28:e93466. doi: 10.2196/93466 (PMC13484949; doi:10.2196/93466)
Supplement: Supplementary file 12 [file jmir-v28-e93466-s012.docx]

| **AI classification outcome** | **Clinical interpretation** | **Assumed clinician response** | **Potential safety risk** | **Risk mitigation assumption** |
| --- | --- | --- | --- | --- |
| True positive | Patients are correctly identified as high risk | Earlier clinician review, bedside reassessment, closer monitoring, ICU team notification, and escalation of care when clinically appropriate | Excessive dependence on AI output or premature closure | Human oversight, protocol-based response, clinician confirmation, and documentation of alert response |
| False positive | Patients receive a high-risk alert without subsequent deterioration | Clinical reassessment without automatic treatment change | Alert fatigue, unnecessary workload, unnecessary testing, or reduced clinician responsiveness over time | Threshold governance, periodic review of alert burden, local workflow adjustment, and feedback-based refinement |
| False negative | Patients are not alerted despite high-risk status | Usual care and routine monitoring continue | Delayed recognition if clinicians over-rely on the absence of an alert | AI does not replace standard care; routine surveillance, escalation pathways, and clinician judgment remain primary |
| True negative | Patients are correctly classified as lower risk | Routine care with periodic reassessment | Over-reassurance or reduced vigilance | Periodic reassessment continues according to standard protocols; clinicians retain final decision-making authority |

**Note**. This table summarizes the workflow assumptions used to clarify human-AI interaction in the economic model. The AI system was conceptualized as a clinician-facing clinical decision support system rather than an autonomous diagnostic or treatment system. Safety risks and mitigation assumptions were informed by prior literature on clinical decision support systems, alert fatigue, automation bias, patient safety, and human oversight in AI-assisted healthcare.
