# Supplementary material for: Value of AI in Critical Care Using Real-World Evidence on Intensive Care Unit Mortality Prediction: Cost-Utility Analysis
Source: J Med Internet Res. 2026 Aug 18;28:e93466. doi: 10.2196/93466 (PMC13484949; doi:10.2196/93466)
Supplement: Supplementary file 13 [file jmir-v28-e93466-s013.docx]

**Note**. This figure presents the net monetary benefit of AI-assisted ICU mortality prediction and usual care across alternative AI implementation cost assumptions. The vertical line indicates the estimated AI cost threshold of KRW 6,531,070 per monitored admission, at which the net monetary benefit of the AI-assisted strategy equals that of usual care. Below this threshold, the AI-assisted strategy remains cost-effective at a willingness-to-pay threshold of KRW 40 million per QALY. Costs are presented in 2023 KRW; approximate USD equivalents can be calculated using KRW 1,350=USD 1.
