# Supplementary material for: Value of AI in Critical Care Using Real-World Evidence on Intensive Care Unit Mortality Prediction: Cost-Utility Analysis
Source: J Med Internet Res. 2026 Aug 18;28:e93466. doi: 10.2196/93466 (PMC13484949; doi:10.2196/93466)
Supplement: Supplementary file 14 [file jmir-v28-e93466-s014.docx]

CHEERS-AI reporting checklist for the cost-utility analysis of AI-assisted ICU mortality prediction

| **Section/topic** | **No.** | **Guidance for reporting** | **AI elaboration** | **Reported in section** |
| --- | --- | --- | --- | --- |
| **Title** | | | | |
| Title | 1 | Identify the study as an economic evaluation and specify the interventions being compared. | Indicate that the intervention involves an AI component that is under evaluation. | Title: “Value of Artificial Intelligence in Critical Care: A Cost-Utility Analysis with Real-World Evidence on ICU Mortality Prediction” |
| **Abstract** | | | | |
| Abstract | 2 | Provide a structured summary that highlights context, key methods, results, and alternative analysis. | Specify the purpose of the intervention with an AI component, and the AI technique used. | Abstract: Background, Objective, Methods, Results, and Conclusions. The abstract identifies the intervention as an AI-based mortality prediction model for ICU patients and reports the use of a Markov model, ICERs, QALYs, deterministic sensitivity analysis, and probabilistic sensitivity analysis. |
| **Introduction** | | | | |
| Background and objectives | 3 | Give the context for the study, the study question, and its practical relevance for decision making in policy or practice. |  | Introduction: describes the need for AI-based mortality prediction in ICU care, limited evidence on downstream clinical and economic outcomes, and the objective of evaluating the potential cost-effectiveness of AI-assisted ICU mortality prediction in Korea. |
| **Methods** | | | | |
| Health economic analysis plan | 4 | Indicate whether a health economic analysis plan was developed and where available. |  | Methods – Study design. No separate health economic analysis plan was prospectively registered. |
| Study population | 5 | Describe characteristics of the study population (such as age range, demographics, socioeconomic, or clinical characteristics). |  | Methods – Model structure. Target population consisted of hypothetical Korean adults aged ≥19 years admitted to the ICU. Demographic characteristics and age-stratified inputs were derived from NHIS claims data. |
| Setting and location | 6 | Provide relevant contextual information that may influence findings. |  | Methods – Model structure; AI-Specific Reporting and Implementation Assumptions. Korean healthcare system, hospital ICU settings, payer perspective. |
| Comparators | 7 | Describe the interventions or strategies being compared and why chosen. | Describe key details of the AI component of the intervention (and comparators, if appropriate), including: a) the classification by intended purpose and risk tier (for digital health technologies); b) the AI technique used; c) whether it is “locked” (static) or adaptive; d) the version under evaluation; e) the purpose of the intervention, including its potential impact on care; f) the intended user(s), and how users interact with it; g) additional requirements to use it; h) how it is expected to provide benefit over the standard of care. | Methods – Clinical Workflow and Safety Assumptions; AI-Specific Reporting and Implementation Assumptions. Comparison between AI-assisted care and usual care. AI intervention described as a machine-learning–based clinical decision support system (CDSS) deployed in ICU settings. Intended users were clinicians, with AI alerts supporting risk stratification and clinical review. Model version, threshold assumptions, implementation requirements, and expected benefits over usual care are described. |
| User autonomy | AI 1 | Indicate whether the AI intervention (and comparators, if appropriate) is directive, or whether the user(s) retains autonomy to make the care decision. |  | Methods – Clinical Workflow and Safety Assumptions. The AI system operated under human oversight; clinicians retained final responsibility for interpretation and management decisions. AI alerts were intended to support, not replace, clinical decision-making. |
| Perspective | 8 | State the perspective(s) adopted by the study and why chosen. |  | Methods – Model structure. Analysis conducted from the Korean payer perspective. |
| Time horizon | 9 | State the time horizon for the study and why appropriate. |  | Methods – Model structure; Scenario analyses. Lifetime Markov model with additional 5-year and 10-year scenario analyses. |
| Discount rate | 10 | Report the discount rate(s) and reason chosen. |  | Methods – Utility values; Key outcome measures. Costs and QALYs were discounted at an annual rate of 4.5%, consistent with Korean health economic evaluation practice. Because the model used monthly cycles, the annual discount rate was converted to a monthly cycle-specific discount factor and applied to both costs and QALYs. |
| Selection of outcomes | 11 | Describe what outcomes were used as the measure(s) of benefit(s) and harm(s). | Describe whether the measure(s) chosen to indicate the benefits and harms of the AI intervention (and comparators) relates to health outcomes, diagnostic outcomes, process outcomes, or other/multiple outcomes. | Methods – Utility values; Key outcome measures. Outcomes included QALYs, ICERs, NMB, diagnostic performance parameters, and mortality reduction assumptions. Benefits relate to health outcomes and process outcomes. |
| Measurement of outcomes | 12 | Describe how outcomes used to capture benefit(s) and harm(s) were measured. | For model-based analysis, describe any assumptions used to inform the potential benefit(s) and harm(s) of the AI intervention in the model (and comparators, if appropriate). Describe the plausibility of analyst assumptions, citing any supportive evidence. | Methods – Clinical effectiveness inputs; Utility values; Key outcome measures. Outcomes measured using NHIS claims data, published utility estimates, mortality hazard ratios, and diagnostic performance parameters. Assumptions regarding AI-related mortality reduction and clinician-mediated benefit pathways are described and supported by literature. |
| Measurement of AI effect | AI 2 | Describe the data sources (assessment studies) for the AI intervention's impact on outcomes. |  | Methods – Clinical effectiveness inputs; Supplementary Box S2. Diagnostic performance was based on the published real-world implementation study, and the mortality reduction effect was modeled using a proxy HR from prior ICU/intensivist-supported care literature. |
| Measurement of AI learning over time | AI 3 | If the AI intervention (and comparators, if appropriate) learns over time, explain how this affects its performance at the individual level and how this was measured. |  | Not applicable. The AI system was modeled as a fixed/locked model; no adaptive learning over time was assumed. |
| Development of AI component | AI 4 | Describe how the AI component of the intervention (and comparators, if appropriate) was developed, including the training data used and how errors and biases were identified, or cite a source that provides this information. |  | Supplementary Box S2. Development details of the AI early warning system were cited from the published implementation study; the checklist refers readers to that source for information on model development, data source, and performance evaluation. |
| Validation of AI component | AI 5 | Describe how the AI component of the intervention (and comparators, as appropriate) and its performance estimates were validated, or cite a source that provides this information. |  | Supplementary Box S2. The reference real-world implementation study reported validation/performance estimates for the deployed AI early warning system, including AUROC, AUPRC, sensitivity, and specificity at the selected threshold. |
| Health benefit | AI 6 | Describe how the AI intervention (and comparators, if appropriate) could directly or indirectly provide a health benefit. |  | Introduction; Methods – Clinical Workflow and Safety Assumptions; Discussion – Interpretation of Findings. The AI intervention was expected to provide indirect health benefit by supporting earlier recognition of high-risk patients, clinician review, closer monitoring, and timely escalation of care. |
| Population differences | AI 7 | Describe important differences between the data sources (assessment studies) for the AI intervention's impact on outcomes and the data set that was used to develop the AI intervention (training data set). |  | Supplementary Box S2; Limitations. The diagnostic performance source included hospitalized patients in general wards and ICUs, whereas the economic model focused on Korean adult ICU patients; this difference was discussed as a limitation affecting generalizability. |
| Valuation of outcomes | 13 | Describe the population and methods used to measure and value outcomes. |  | Methods – Utility values. QALYs were calculated using utility weights for ICU admission, general ward hospitalization, post-discharge survival, and death, based on published utility estimates. |
| Measurement and valuation of resources and costs | 14 | Describe how costs were valued. | Describe the purchase cost of the AI intervention (and comparators, if appropriate) and what it is composed of. Describe any additional implementation and maintenance costs. | Methods – Cost input; Table 1; Table 2. Costs were estimated from NHIS claims data and expressed in 2023 KRW. AI implementation costs included infrastructure, licensing, maintenance, technical support, staff training, and operational management. |
| Currency, price date, and conversion | 15 | Report the dates of the estimated resource quantities and unit costs, plus the currency and year of conversion. |  | Methods – Cost input. Costs were reported in 2023 Korean won, with selected values converted to US dollars using KRW 1,350 = USD 1. |
| Rationale and description of model | 16 | If modeling is used, describe in detail and why used. Report if the model is publicly available and where it can be accessed. | Describe if the AI component of the intervention has influenced the choice of health economic model and explain why. | Methods – Study design; Model structure; Figure 1. A state-transition Markov model was used to compare AI-assisted care with usual care and included ICU, general ward, post-discharge well, and death states. |
| Analytics and assumptions | 17 | Describe any methods for analyzing or statistically transforming data, any extrapolation methods, and approaches for validating any model used. |  | Methods – Clinical effectiveness inputs; Sensitivity analysis; Supplementary Box S1. The model used NHIS-derived transition probabilities, published utility estimates, diagnostic performance inputs, and mortality reduction assumptions; uncertainty was assessed using DSA, PSA, and scenario analyses. |
| Modeling of AI learning over time | AI 8 | If the AI intervention (and comparators, if appropriate) learns over time at the individual level, describe any assumptions used to model how this learning affects its performance over time. |  | Not applicable. The AI system was modeled as a fixed/locked system, and no learning-related performance changes over time were modeled. |
| Characterizing heterogeneity | 18 | Describe any methods used for estimating how the results of the study vary for subgroups. |  | Methods – Model structure; Table 2; Real-world analysis using NHIS data. Age-stratified transition probabilities, hospitalization costs, and survival outcomes were estimated from NHIS claims data. |
| Characterizing distributional effects | 19 | Describe how impacts are distributed across different individuals or adjustments made to reflect priority populations. |  | Not performed. Distributional cost-effectiveness or equity-weighted analysis was not conducted. |
| Characterizing uncertainty | 20 | Describe methods to characterize any sources of uncertainty in the analysis. |  | Methods – Sensitivity analysis. Parameter and structural uncertainty were assessed using one-way deterministic sensitivity analysis, probabilistic sensitivity analysis with 100,000 Monte Carlo simulations, and scenario analyses. |
| Approach to engagement with patients and others affected by the study | 21 | Describe any approaches to engage patients or service recipients, the general public, communities, or stakeholders (such as clinicians or payers) in the design of the study. |  | Not performed. No formal patient, public, or stakeholder engagement was conducted for the study design. |
| **Results** | | | | |
| Study parameters | 22 | Report all analytic inputs (such as values, ranges, and references) including uncertainty or distributional assumptions. |  | Methods – Table 1 and Table 2; Supplementary Box S1. All major model inputs, including diagnostic accuracy, mortality reduction, AI implementation cost, utility values, transition probabilities, medical costs, ranges, distributions, and data sources, were reported. |
| Summary of main results | 23 | Report the mean values for the main categories of costs and outcomes of interest and summarize them in the most appropriate overall measure. |  | Results – Base-case analysis; Table 3. Mean costs, QALYs, incremental costs, incremental QALYs, ICER, and NMB were reported for AI-assisted care versus usual care. |
| Effect of uncertainty | 24 | Describe how uncertainty about analytic judgments, inputs, or projections affect findings. Report the effect of choice of discount rate and time horizon, if applicable. |  | Results – Sensitivity analysis; Figures 2–4; Supplementary Tables S5–S6. Uncertainty was assessed using PSA, DSA, CEAC, incremental cost-effectiveness scatterplot, tornado diagram, and scenario analyses with alternative mortality effects and time horizons. |
| Impact of AI uncertainty | AI 9 | Indicate the extent to which features of the AI intervention may contribute to increased uncertainty about its cost-effectiveness. |  | Results – One-way sensitivity analysis; Discussion – Limitations. AI-specific uncertainty was reflected through diagnostic performance parameters, especially true-positive rate, AI implementation cost, false-negative assumptions, alert burden, and workflow-related implementation uncertainty. |
| Effect of engagement with patients and others affected by the study | 25 | Report on any difference patient/service recipient, general public, community, or stakeholder involvement made to the approach or findings of the study. |  | Not applicable. Patients, caregivers, and other stakeholders were not involved in study design. |
| **Discussion** | | | | |
| Study findings, limitations, generalizability, and current knowledge | 26 | Report key findings, limitations, ethical or equity considerations not captured, and how these could affect patients, policy, or practice. | Comment on potential biases associated with the AI intervention (eg, algorithmic bias) and implications for the generalizability and interpretation of results (eg, reinforcing existing health inequalities). | Discussion – Principal Findings, Interpretation of Findings, Policy and Implementation Implications, and Limitations. The manuscript reports key findings, compares them with prior literature, discusses workflow risks including alert fatigue and automation bias, and notes limitations related to diagnostic performance, generalizability, post-ICU outcomes, and small incremental QALY gain. |
| Implementation of AI | AI 10 | Comment on any requirements needed to integrate the AI intervention (and comparators, as appropriate) into practice, and other implementation considerations relating to the AI component of the intervention, including implications for the interpretation of cost-effectiveness results. |  | Discussion – Policy and Implementation Implications; Limitations. The manuscript discusses workflow integration, clinician response, alert governance, local implementation cost, continuous monitoring, staff training, alert fatigue, automation bias, and interpretation of cost-effectiveness results under implementation uncertainty. |
| Source of funding | 27 | Describe how the study was funded and any role of the funder in the identification, design, conduct, and reporting of the analysis. |  | Declarations – Funding. The study reports research support from aitrics Corp and states that the sponsor had no role in the design of the health economic evaluation, NHIS data access, statistical analysis, Markov model development, model coding, interpretation, manuscript writing, or submission decision. |
| Conflicts of interest | 28 | Report authors conflicts of interest according to journal or International Committee of Medical Journal Editors requirements. |  | Declarations – Competing interests; Data Access and Analytic Independence. The manuscript discloses aitrics-affiliated authors and sponsor involvement, and describes independent analysis, restricted NHIS data access, lack of sponsor control over model code and interpretation, and absence of direct use of proprietary patient-level commercial data. |

Note. Pre-existing CHEERS 2022 items are numbered 1 through 28. Elaborations that add AI-specific context are shown to the right of the corresponding CHEERS 2022 item. New, AI-specific extension items are numbered “AI 1-10.” AI indicates artificial intelligence; CHEERS-AI, Consolidated Health Economic Evaluation Reporting Standards for Interventions that use AI.
